# HeT-A_pi1, a piRNA Target Sequence in the Drosophila Telomeric Retrotransposon HeT-A, Is Extremely Conserved across Copies and Species

**DOI:** 10.1371/journal.pone.0037405

**Published:** 2012-05-21

**Authors:** Natalia Petit, David Piñeyro, Elisenda López-Panadès, Elena Casacuberta, Arcadi Navarro

**Affiliations:** 1 Departament de Ciències Experimentals i de la Salut (DCEXS), Universitat Pompeu Fabra, Barcelona, Spain; 2 Institut de Biologia Evolutiva (CSIC-UPF), Barcelona, Spain; 3 National Institute for Bioinformatics (INB), Population Genomics Node, Universitat Pompeu Fabra, Barcelona, Spain; 4 Institució Catalana de Recerca i Estudis Avançats (ICREA), Catalonia, Spain; Institut de Biologia Evolutiva - Universitat Pompeu Fabra, Spain

## Abstract

The maintenance of the telomeres in *Drosophila* species depends on the transposition of the non-LTR retrotransposons *HeT-A*, TAHRE and *TART*. *HeT-A* and *TART* elements have been found in all studied species of *Drosophila* suggesting that their function has been maintained for more than 60 million years. Of the three elements, *HeT-A* is by far the main component of *D. melanogaster* telomeres and, unexpectedly for an element with an essential role in telomere elongation, the conservation of the nucleotide sequence of *HeT-A* is very low. In order to better understand the function of this telomeric retrotransposon, we studied the degree of conservation along *HeT-A* copies. We identified a small sequence within the 3′ UTR of the element that is extremely conserved among copies of the element both, within *D. melanogaster* and related species from the *melanogaster* group. The sequence corresponds to a piRNA target in *D. melanogaster* that we named HeT-A_pi1. Comparison with piRNA target sequences from other Drosophila retrotransposons showed that HeT-A_pi1 is the piRNA target in the *Drosophila* genome with the highest degree of conservation among species from the *melanogaster* group. The high conservation of this piRNA target in contrast with the surrounding sequence, suggests an important function of the HeT-A_pi1 sequence in the co-evolution of the HeT-A retrotransposon and the *Drosophila* genome.

## Introduction

The function of eukaryotic telomeres goes beyond capping the end of the DNA molecule and has been found of key importance for other cellular processes such as senescence, genomic stability and oncogenesis [1]–[3]. Telomeres from eukaryotic chromosomes consist in arrays of repeated sequences that in most eukaryotes are maintained by the telomerase holoenzyme [4]. In contrast, in the Drosophila genus telomeres are maintained by a different mechanism. Telomere-specific retrotransposons are reverse transcribed specifically onto the end of the chromosomes [3], [5], [6]. *Drosophila* species contain two main types of retroelements in their telomeres, *TART* and *HeT-A,* with a few copies of *TAHRE* inside the melanogaster group [7]. These two retrotransposons are non-LTR retroelements with features that distinguish them from other non-telomeric elements. *HeT-A* and *TART* have very long 3′ untranslated regions (UTR) and are specifically targeted to the end of the chromosomes, thus maintaining telomere length [6]. *HeT-A* is the main component of *D. melanogaster* telomeres and its orthologues have been described in almost all telomeres of studied *Drosophila* species, from *D. melanogaster* to *D. virilis*, indicating that the function of this element predates the Drosophila genus [8]–[12].

The invasion of eukaryote genomes by transposable elements (TEs) triggered the development of a great diversity of defense mechanisms. These defense mechanisms are actively evolving to control transposition at different levels. In recent years small interfering RNAs have been highlighted as a very powerful mechanism of gene regulation. Piwi-interacting RNAs (piRNAs) [13], [14] are a particularly interesting class of RNAs. They act mainly upon TEs in germ line tissues where control of transposition is critical, because new transpositions would be passed to the offspring. Two versions of the piRNA pathway exist in ovaries depending on whether the cell belongs to the oocyte and the accompanying nurse cells (germ cells) or to the follicle cells that surround the egg (somatic cells; [15]).The generation of piRNAs in germ cells by the action of the different Piwi (P-element induced wimpy testis) proteins, Aubergine, Argonaute3 and Piwi, is dependent on an amplification cycle, the so called Ping-Pong cycle. In that cycle, a primary piRNA complementarily recognizes its target and recruits PIWI-proteins, which will cleave the transcript generating a secondary piRNA, which, in turn, will further amplify the process. According to a recent classification based on the relative abundances of sense and antisense piRNAs loaded into the different PIWI-proteins TEs can be classified in three groups, *HeT-A* belongs to Group I [16], [17]. Group I transposons are heavily repressed in germ line cells and present a strong *ping-pong* signature that derives from a 10 nucleotide overlap between antisense piRNAs bound to Aubergine and sense piRNAs bound to Ago3 [17].

Whatever the exact mechanism of action of piRNAs, processing of the transposon’s mRNA results in effective posttranscriptional silencing and further amplifies the presence of sense and antisense piRNAs for that particular copy of the TE. These newly generated piRNAs can now target both strands of a genomic copy of a TE and direct specific silencing complexes to remodel the chromatin environment achieving transcriptional silencing [14], [18]. The fact that piRNAs can silence TEs provides an opportunity for an arms race in which natural selection would favor transposable elements that escape this kind of control by acquiring mutations in their piRNA target sequences. This process tends to make piRNAs rapidly evolving sequences [19], [20].

Beyond their functional role in the control of transposable elements, piRNAs may also have regulatory roles in heterochromatin assembly and epigenetic regulation [19], [21]. The complexity of small-RNA mediated epigenetic regulation in higher organisms remains largely unexplored, and it has been suggested that the interaction between PIWI-proteins and piRNAs could promote heterochromatin or euchromatin formation depending on the chromatin context in *D. melanogaster* [21], [22]. In addition, it has been recently shown that two components of the PIWI pathway, *Armitage* and *Aubergine* are required for the proper assembly of the telomere protection complex demonstrating additional functional roles of this pathway besides TEs silencing [23].

Although data available for the heterochromatic portion of the *Drosophila* genomes is far from complete, different studies indicate a lack of sequence conservation in the nucleotide sequence of *HeT-A* indicating a fast evolution of this retroelement [6], [8]–[11], [24], [25]. This seems contradictory for a transposable element with an important function in the stem cells maintenance and raises questions about the co-evolution of telomeric transposons and *Drosophila* telomeres. Motivated by this contradiction, we studied the degree of conservation along the copies of *HeT-A* looking for putative regulatory sequences. We identified a small sequence within the 3′ UTR of the element that is highly conserved among copies and species from the *melanogaster* group (5–15 MY of genetic distance). Further analysis of this sequence revealed that, quite surprisingly, it corresponds to a piRNA target sequence. Because of the highly variable nature of *HeT-A* sequence, the extreme conservation of this piRNA target sequence, HeT-A_pi1, suggests a possible role in the co-evolution of *HeT-A* and the *Drosophila* genome.

## Results

### Sequence Conservation at the 3′UTR of the HeT-A Retrotransposon and Presence of piRNA Targets

**Figure 1:**
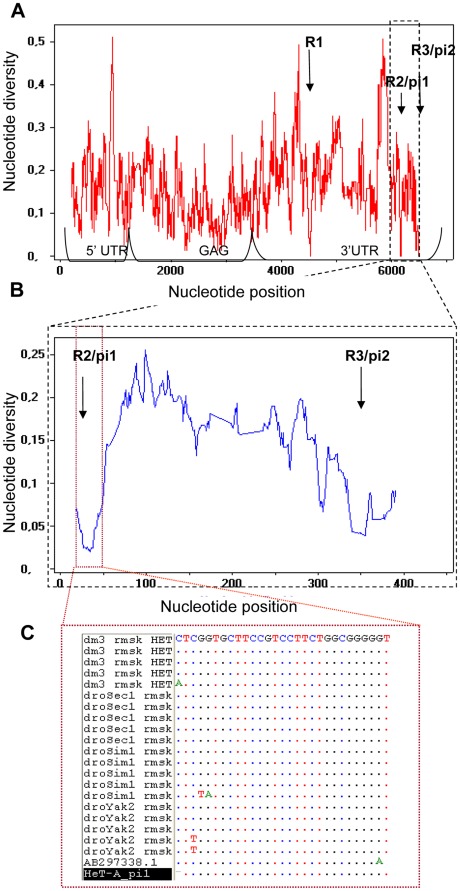
Nucleotide diversity estimates among elements and species. (**A**) Conservation analysis of the six complete *HeT-A* elements from *D.* *melanogaster*. Sliding windows of 25 ntds size and 1 ntd steps are represented. The significantly conserved regions (R) in the 3′UTR are marked with arrows. (**B**) Conservation analysis of the last 500 ntds of the 3′UTR among species (*D. melanogaster, D. sechellia, D. simulans, D. yakuba*). Graph constructed with an alignment of homologous sequences, longer than 350 ntds, obtained from the Blast analysis of the 3′ UTR sequence with a window size of 25 ntds and step size of 1 ntds (see Methods and Results S1). Number of aligned sequences: *D. melanogaster* 26, *D. sechellia* 26, *D. yakuba* 6 and *D. simulans* 12. The estimated average nucleotide diversity among all 72 sequences is 0.13571. For nucleotide diversity within each species see Figure S1. (**C**) Alignment of the piRNA target sequence among *HeT-A* copies from four Drosophila species. Nucleotide diversities: all, 0.048; *D. sechellia*, 0.028, *D. yakuba*, 0.021; *D. simulans*, 0.058; and *D. melanogaster*, 0.0437 (See Figure S1).

To search for putative functional elements along the *HeT-A* retrotransposon, we performed two analyses. First we investigated the levels of sequence conservation amongst the six described *D.* *melanogaster* complete *HeT-A* copies [6]. We measured nucleotide diversity as the average number of pairwise nucleotide differences among copies of the element using sliding windows of several sizes (see Methods and Results S1). In Figure 1A, a sliding-window graph (25 ntds long, with a 1 ntds step) shows that there are three small conserved regions, R1, R2 and R3, in the 3′ UTR of the element. Conservation analyses performed with DNAspv5 [26] using a range of different windows and conservation thresholds; indicated that only these three small regions are fully conserved (conservation = 1) across copies for all the window sizes used, indicating that these three regions are more conserved than any other fragment of the element. They are even more conserved than any fragment of the *gag* coding region, which in average is, of course, the most conserved part of the element (Figure 1A and Table S1).

Our second analysis considered interspecific variability across all the available genomes of Drosophila species. We performed a Blast analysis of the three functional parts (3′ UTR, 5′UTR and *gag* coding region) of a randomly selected *HeT-A* copy from *D.* *melanogaster* (*HeT-A*{}6268) against *HeT-A* databases comprised by the *HeT-A* sequences of the eleven Drosophila genomes annotated by RepeatMasker at the UCSC Genome Bioinformatics website (http://genome.ucsc.edu/; See Methods). For the *gag* coding regions, we only observed homology with the closest species (*D.* *sechellia* and *D. simulans*, <5 Myr divergence). In contrast, for the 3′ UTR region we also obtained homology hits in *HeT-A* copies of *D. yakuba*. (∼10 Myrs divergence, see TableS2). Since the 3′ UTR hits were located in its last 500 bp, where the R2 and R3 regions are located, for these 500 bp we extended the sliding window analysis explained above to the three Drosophila species presenting homology hits (*D. sechellia*, *D. simulans* and *D. yakuba*). Two out of the three regions (R2 and R3) were found to be significantly conserved (Figure 1B, full results and details are given in Methods and Results S1).

In order to explain the conservation of the R2 and R3 regions, we performed several analyses looking for a function for these sequences. First, we checked whether these regions are part of the sense or antisense *HeT-A* promoters. From the known functional annotation of *HeT-A*, we could exclude that the R2 region was part of the antisense promoter [27] and, although included in the sense promoter, the R2 is not necessary for driving transcription [28] (Figure S2). Second, we blasted the final 500 ntds of the 4R{}6268 *HeT-A* element, which span the R2 and R3 conserved regions, against the full NCBI Nucleotide Database (http://blast.ncbi.nlm.nih.gov/Blast.cgi). Besides *HeT-A* sequences, several matches were obtained corresponding to two mapped *D.* *melanogaster* cDNAs (BT015972 and BT030306) and one antisense piRNA from *D. melanogaster* testes (AB297338.1; see Methods and Results S1). The high identity of the R2 conserved region with the antisense piRNA sequence AB297338.1 (27 out 28 identical nucleotides, see also Figure 1C) suggests that corresponds to the target sequence for this piRNA.

Given that a piRNA had been discovered, we searched for other possible target sequences of this piRNA besides *HeT-A*. With this purpose we blasted the piRNA sequence looking for matches of at least 24 nucleotides against a database containing all described TEs (N = 176 from Flybase) and a database containing all mRNAs from *D. melanogaster*. We detected 47 mRNAs carrying the sense or antisense sequence of the piRNA (see Table S4). Inspection of the sequence of these mRNAs showed that these are fully composed by repetitive sequences from different transposable elements including *HeT-A*. These mRNAs map in heterochromatic unassembled parts of the genome, without any known genes, and they could be transcripts from piRNA clusters [16], [17]. Putting all this evidence together, it seems clear that this piRNA does not target another TE sequence or known gene different from *HeT-A.*

In order to ascertain whether other conserved piRNA targets could be found along the *HeT-A* sequence. We split the six known *HeT-A* complete copies in 30 ntds fragments with an overlap of 29 ntds (i.e. a 1ntds step). These fragments were blasted against the database of small RNA reads obtained by Li et al. [17] from wild-type ovaries of *D. melanogaster* (SRX002245). We obtained a total of 21,319 different small RNAs reads that matched one or more fragments of the different *HeT-A* copies. These reads represent 7,048 putative piRNA sequences matching different *HeT-A* sequences in *D. melanogaster*, (42% sense and 58% antisense). Most of these piRNA targets are located within the *gag* coding region (Table 1 and Methods and Results S1) producing a negative correlation between nucleotide diversity and number of piRNAs targets (see Methods and Results S1, Table S3, Figures S3,S4,S5,S6,S7,S8). Although many RNA reads target moderately conserved regions of the *HeT-A* copies, more than 50% of these reads exactly match only a single copy of the element and only 17 RNA reads match fragments coming from all six complete *HeT-A* copies (see Figure S8 and Table 1). This frequency distribution of target piRNAs along *HeT-A* agrees with the expectations from an arms-race between the host and the controlled TEs [29].

**Table 1:** Number of piRNA reads, along the *HeT-A* copies and coordinates for piRNA targets HeT-A_pi1 and 2 in each described copy from *D. melanogaster.*

			piRNA coordinates			
HeT-A copies name	Number of reads	Number of differentpiRNAs (Total/gag coding region)	HeT-A_pi1 sense	HeT-A_pi1anti-sense	HeT-A_pi2 sense	HeT-A_pi2anti-sense
HeT-A{}6265	6551	1318/850	5496.5524	5506.5529	5821.5847	5821.5844
HeT-A{}6274	6725	1307/829	5481.5509	5491.5514	5806.5833	5806.5829
HeT-A{}4800	4585	1057/644	5645.5673	5655.5680	5974.6001	5974.5997
HeT-A{}6268	5506	1099/724	5651.5679	5661.5686	5980.6007	5980.6003
HeT-A{}6262	5675	1169/786	5431.5459	5441.5446	5760.5786	5760.5783
HeT-A23Znk	6952	1098/786	5728.5756	5737.5762	6048.6075	6048.6071

The small 17 RNA reads found to target all six *D. melanogaster HeT-A* copies correspond to sense and antisense RNA sequences targeting precisely the two conserved regions in the last 500 nucleotides of the 3′UTR, R2 and R3 (Table 1 and Figure 1A). At this point we have evidence for the two regions being targeted by piRNA, since piRNAs matching these target sequences have been found to bind PIWI-proteins, (Piwi and Aubergine) in databases of piRNAs obtained by Li et al. [17] (supporting R2 and R3; SRX002242-3) and Nishida et al. [30] (supporting R2; AB297338.1). Therefore, we renamed the R2 conserved region as piRNA target HeT-A_pi1 and R3 as piRNA target HeT-A_pi2. Coordinates of these piRNAs in each *HeT-A* copy are presented in Table 1. Figure S2 shows the relative positions of HeT-A_pi1 and HeT-A_pi2 relative to known functional features of the 3′UTR.

### The piRNA Target HeT-A_pi1 is Conserved within the 3′UTR of HeT-A Orthologues from Different Drosophila Species

To ascertain up to which level the sequence and position of the two *HeT-A* piRNA targets (HeT-A_pi1 and HeT-A_pi2) had been conserved across the evolution of the whole Drosophila group, we investigated the presence of these piRNA targets and their flanking regions in related species from the melanogaster group (*D.* *melanogaster*, *D. simulans*, *D. sechellia* and *D. yakuba*) for which we had detected *HeT-A* copies with the conserved regions (see Methods and Results S1). To do so, we again used our *HeT-A* databases (see Methods), but in order to ensure that the piRNA targets were located within the same region of the element, that is in order to ensure we were dealing with orthologous piRNA targets, instead of using only the HeT-A_pi1 sequence we also blasted a 250 ntds-long sequence (“homologous sequence”) starting 100 ntds 5′ before the piRNA target sequence. The piRNA target HeT-A_pi2 was only found in *D. melanogaster*, while HeT-A_pi1 was found in all four species: *D. melanogaster, D.* *simulans*, *D. sechellia* and *D. yakuba*. Thus, in what follows we focused in HeT-A_pi1 (region R2). The numbers of hits obtained by the sense piRNA HeT-A_pi1 and the 250 nucleotides sequence in each species are summarized in Table 2. The results for the antisense piRNA HeT-A_pi1 are nearly identical since this pair overlaps in nearly 20 nucleotides. More than 80% of the homologous *HeT-A* sequences contain the sequence of the piRNA HeT-A_pi1, with at least 24 consecutive identical nucleotides. The nucleotide alignment shown in Figure 1C further illustrates the conservation of that piRNA in different species.

**Table 2:** Summary of hits from Blast analyses of the HeT-A_pi1 (Number of hits piRNA) and the 250 ntds sequences (Number of hits TE) containing the sequence of HeT-A_pi1 against databases of annotated *HeT-A* sequences in different *Drosophila* species.

Species	Number hits piRNA	Number of hits TE (A)	Number of shared hits*(B)	%TE seqs. with the piRNA((B/A)*100)
*D. sechellia*	33	32	29	90.63
*D. simulans*	12	14	12	85.71
*D. yakuba*	59	40	39	97.50
*D. melanogaster*	34	41	33	80.49

* number of hits where the 250 ntds sequences contain the piRNA HeT-A_pi1.

To further quantify the conservation of the HeT-A_pi1 target sequence and to ensure that conservation was due to the HeT-A_pi1 target sequence itself, and not to the surrounding 250 ntds window, we used a log-likelihood ratio test (*see Methods*) to check whether variability of the piRNA target HeT-A_pi1 (among copies and within species) was significantly lower than the nucleotide variability of the 250 ntds window flanking HeT-A_pi1 sequence in *D. melanogaster* (p = 0.013), *D. sechellia* (p = 0.009) and *D. yakuba* (p = 0.038). In *D. simulans*, where both the number of homologue copies and nucleotide diversity are lower than for the other three species the test is not significant (Table 2 and supp. Table S7).

### Putative Functional and Transcribed Elements Contain the piRNA Target HeT-A_pi1

As mentioned above, all the six complete elements in *D.* *melanogaster* contain the exact target sequence for the HeT-A_pi1 piRNA within their 3′UTRs (Table 1). In order to know if the conserved HeT-A_pi1 is also found in putative functional elements in other species, an *in silico* analysis searching for complete and putative functional *gag* coding regions in the *HeT-A* databases of the different species was performed. The sequence of the Gag protein was t-blasted against the *HeT-A* databases of the different species. Because the nucleotide sequence identity among *gag* coding regions with the most distant species, *D. yakuba*, is low (see Methods and Results S1 and Table S2) we used two different Gag protein sequences. The Gag protein from *D. melanogaster*’s *HeT-A*{}6268 copy was blasted against *D. sechellia* and *D. simulans*; while the sequence from the previously described *HeT-A* orthologue in *D. yakuba* (AF043258; [24]) was blasted against the *D. yakuba*. Analysis revealed eleven putatively functional *gag* coding regions (nine in *D. sechellia,* one in *D. simulans* and one in *D. yakuba*; Table 3). The sequence of the target HeT-A_pi1, was searched within a 5 kb window downstream of the identified *gag* coding regions. In all cases where genomic sequence was available, the piRNA target HeT-A_pi1 was found (Table 3).

**Table 3:** Coordinates of complete *gag* coding regions and HeT-A_pi1 targets in different Drosophila species. Nucleotide changes in the piRNA target sequence labeled in red, otherwise perfect identity.

Species	*gag* gene coordinates (strand)	piRNA target coordinates	piRNA target sequence
*D. simulans*	chrU:5142940–5145762(+)	chrU:5147652–5147679	tcggtgcttccgtccttctggcgggggt
	super_173∶1481–4303(−)	No downstream sequence	
	super_2∶42799–45513(+)	super_2∶47693–47720	gcggtgctcctgtccttctgatgggggt
	super_986∶2128.4944(−)	No downstream sequence	
	super_296∶1169–3964(+)	super_296∶7345–7372	tcggtgcttccgtccttctggcgggggt
*D. sechellia*	super_182∶4515–7309(−)	super_182∶3079–3106	tcggtgcttccgtccttctggcgggggt
	super_543∶3382–6177(+)	super_543∶7667–7694	tcggtgcttccgtccttctggcggaggt
	super_535∶1469–4282(+)	super_535∶5773–5800	tcggtgcttccgtccttctggcggaggt
	super_330∶1726–4545(−)	No downstream sequence	
	super_173∶1480–4302(−)	No downstream sequence	
*D. yakuba*	chr2R:21104242–21106926(−)	chr2R:21102135–21102162	tcggtgcttccgtccttctggcgggggt

**Figure 2:**
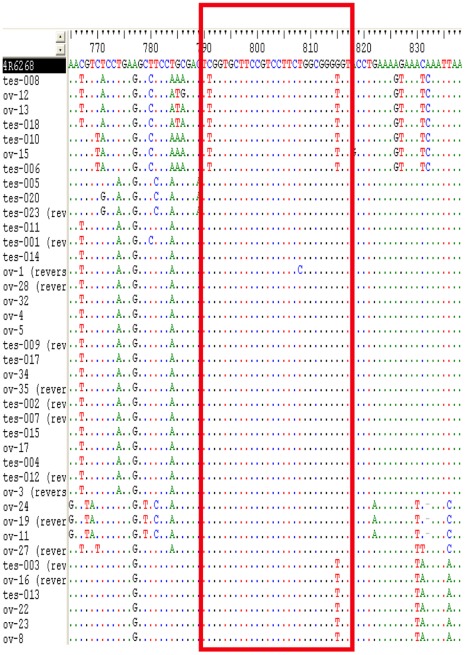
Alignment of 3′UTR transcripts obtained from testes and ovaries of D. melanogaster Oregon R. Nucleotide polymorphisms are indicated. piRNA target HeT-A_pi1 is labelled with a red rectangle.

**Figure 3:**
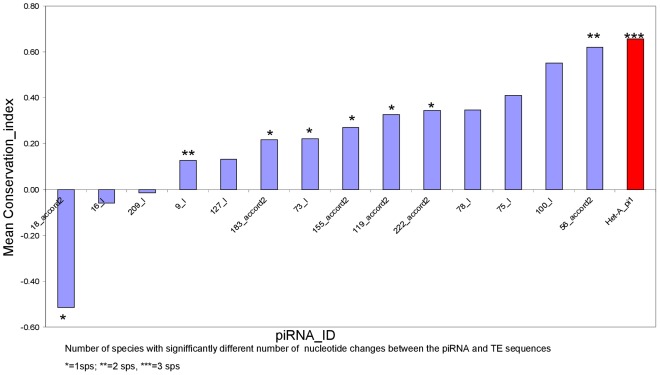
Average conservation index of the fifteen highly conserved piRNA targets from the TEs HeT-A, accord2 and I among D. melanogaster, D. simulans, D. sechellia and D. yakuba species. Asterisks label those cases (species) where the piRNA target sequence has significantly different number of nucleotide changes than the flanking sequence by a log-likelihood ratio test (See Methods). For values within species see Table S7 and Figure S10.

To find out if the sequence of the 3′UTR containing the piRNA is being actively transcribed, we amplified, cloned and sequenced thirty-nine mRNAs of the *HeT-A* 3′UTR from *D. melanogaster* ovaries and testes (strain Oregon-R). All transcripts contain the piRNA target HeT-A_pi1 sequence. The alignment in Figure 2 demonstrates that although the flanking sequence at both sides of the piRNA target shows different nucleotide polymorphisms, variability decreases substantially inside the sequence of the piRNA target (3 changes, nucleotide diversity = 0.028; Figure 3). The sequenced transcripts could be classified in ten groups, seven of which map with more than 97% identity within the arrays of the telomeric elements in the two completely assembled telomeres of *D. melanogaster* (4R and XL, isogenic strain 2057 yellow (*y^1^*); cinnabar (*cn^1^*) brown (*bw^1^*) speck (*sp^1^*)) (see Methods and Results S1 and Table S5). Interestingly, the transcript ov11 is 100% identical to the *HeT-A*{}6274 copy, which maps at the right telomere of chromosome IV. The *HeT-A* copies 23Znk and *HeT-A*{}6274 are the two complete elements with a higher number of small RNA reads (Table 1), which suggests that these two master copies are among the most active in different strains of *D. melanogaster.*

### The piRNA Target HeT-A_pi1 Shows Higher Conservation than Other Target piRNAs in Different TEs

To ascertain whether the case of HeT-A_pi1 is as rare as it seems from the results above, we investigated the level of conservation of piRNAs matching sequences of other transposable elements (TEs) within the *melanogaster* group. We selected the most representative elements of each of the groups defined by Li et al. [17] in order to take into account any possible biological differences in the levels of conservation among these three groups. *Gypsy* from Group III, *Accord2* from Group II and for Group I, the *HeT-A* related elements *Jockey* and *TART* and the *HeT-A* unrelated elements *copia* and *I*. We followed the same strategy that we used for *HeT-A* to find conserved target piRNAs within these retrotransposons in the databases of the four species of the melanogaster group. For each retrotransposon we take 250 piRNAs exactly matching (best hits of at least in 24 consecutive ntds) the canonical TE sequence (see Methods and Results S1 and Figure S9). For each piRNA matching the canonical sequence of each TE we checked for the presence of the piRNA target within a 250 ntds homologous sequence, (beginning 100 ntds. before the piRNA target sequence). This analysis unveiled 98 non-redundant piRNA targets with significant hits and exact match (at least 24 consecutive identical nucleotides) in homologous sequences of the different TEs in all four species (supp. Table S6). Fourteen of these piRNA target sequences are present in more than 50% of the homologous copies and at least in four different copies of the elements (*accord2* and *I*, supp. Table S6).

Since the probability to find the same piRNA target in different copies of one element depends on the level of nucleotide variability of each particular TE, we calculated a conservation index based on the nucleotide diversity of each piRNA target sequence, relative to the nucleotide diversity of the flanking sequence (see Methods). Moreover, because the rate of evolution of transposable elements depends not only on nucleotide substitutions but also on the rate of sequence insertion and deletion [31] we took into account the percentage of TE sequences with the complete target piRNA sequence (Table 4). Figure 3 shows the graphs corresponding to the mean values of the conservation index among species for the fifteen conserved piRNA targets, (see Figure S10 and Table S7 for individual species). Among the fifteen most conserved target piRNAs analyzed, the target HeT-A_pi1 has the highest conservation index and the highest number of species where the sequence variability of the target piRNA is significantly lower than the nucleotide variability of the sequence of the TE without the piRNA target sequence (Figure 3 and Figure S11 and S12).

**Table 4:** Most conserved target piRNAs from the seven analyzed TEs. The values presented are means and standard deviation across species (see Table S7 for individual species analyses).

piRNA_ID	Mean ± SD			
	Nucletide diversity ofpiRNA (A)	Nucleotide diversity ofTE (B)	% TE sequences with thepiRNA (C)	Conservation Index(1-A/B)*C
HeT-A_pi1	0.026±0.010	0.104±0.028	0.886±0.072	0.655976±0.141149
55_accord2	0.017±0.016	0.076±0.095	0.851±0.152	0.641643±0.245810
100_I	0.014±0.013	0.042±0.007	0.789±0.156	0.550814±0.344161
75_I	0.024±0.017	0.045±0.008	0.784±0.173	0.408558±0.412469
222_accord2	0.030±0.022	0.042±0.018	0.717±0.203	0.346673±0.442072
119_accord2	0.022±0.017	0.038±0.012	0.733±0.085	0.344468±0.180259
155_accord2	0.020±0.005	0.040±0.011	0.723±0.128	0.325855±0.110779
78_I	0.024±0.008	0.039±0.009	0.706±0.144	0.269431±0.219061
73_I	0.028±0.019	0.038±0.006	0.729±0.094	0.220935±0.284904
209_I	0.027±0.016	0.039±0.020	0.645±0.029	0.215759±0.225368
9_I	0.025±0.016	0.034±0.009	0.686±0.103	0.132290±0.496102
183_accord2	0.035±0.035	0.036±0.012	0.723±0.142	0.127842±0.413761
127_I	0.033±0.028	0.033±0.010	0.722±0.097	–0.013562±0.561638
18_accord2	0.037±0.010	0.035±0.011	0.651±0.152	–0.058755±0.149838
16_I	0.047±0.015	0.050±0.037	0.746±0.115	–0.513765±1.271019

## Discussion

### Why Would the HeT-A Retrotransposon Conserve a Sequence Containing a piRNA Target?

Our results indicate that the piRNA target HeT-A_pi1 is highly conserved in the different *HeT-A* copies in *D. melanogaster* and also in *HeT-A* orthologues of related species within the melanogaster group: *D. yakuba*, *D. sechellia*, *D. simulans*, presenting a degree of conservation that is even higher than any fragment of the *gag* coding region. Moreover, the piRNA target HeT-A_pi1, is the target piRNA with the highest conservation among 98 conserved target piRNAs in different copies of seven analyzed TEs. Given the high nucleotide variability of the *HeT-A* sequence inside and among species [6], [10], [12], [32] this result strongly suggests that the sequence of this piRNA target, HeT-A_pi1, has been maintained under strong purifying selection for nearly 5–10 million years.

The function of piRNAs has been related to transcriptional and posttranscriptional control of genetic mobile elements [16]–[19], [29], [33]. Although the mechanism of biogenesis and function of these small RNAs is not yet fully understood, the main activities in which piRNAs are involved are aimed to guard genome integrity from the potential deleterious activity of transposable elements. Following an arms-race logic in which the transposable elements would try to escape this control, one would not expect that *HeT-A* would conserve a piRNA target within its sequence, facilitating host control and reducing its capacity to transpose. However, in a series of simulation studies Lu and Clark [29] showed that, under certain circumstances, TEs that are producers and targets of piRNAs have an increased probability of reaching high frequencies or even fixation within populations. These authors suggest that the piRNA sequence provides a “Trojan horse” for retrotransposons, allowing transposition of the elements within a given genome to be fine-tuned. We postulate that the HeT-A_pi1 piRNA could constitute a nice example of Lu & Clark’s “Trojan horse” hypothesis. Because of its telomeric role, the *HeT-A* retrotransposon and the Drosophila genome would have reached an armistice; a sort of symbiosis in which the terminal transposition of the element is allowed when telomere elongation is required, while potentially deleterious transposition events that would result in genomic instability are strongly repressed. We have shown that the transcripts of active copies of the *HeT-A* retrotransposon in germ line tissues (ovaries and testes) carry the piRNA target HeT-A_pi1. We have identified antisense cDNAs (such as BT015972, see Methods and Results S1) that most likely correspond to antisense transcripts produced from the *HeT-A* clusters at the telomeres, which could be sources for antisense HeT-A_pi1 RNAs. Shpiz et al. [27] and Piñeyro et al. [32] have shown that the 3′UTR of the *HeT-A* retrotransposon contains an antisense promoter capable of producing a variety of antisense transcripts from the *HeT-A* clusters at the telomeres. Thus, all the potential factors for the piRNA target HeT-A_pi1 to act as a canonical piRNA target and function to silence the *HeT-A* retrotransposon are undoubtedly in place in *D. melanogaster*.

Interestingly, neither TART nor TAHRE, that are considerably less efficient in successfully transposing into Drosophila telomeres, contain the piRNA target sequence HeT-A_pi1. In fact, a search of the HeT-A_pi1 element in different databases (canonical TEs and mRNAs from *D. melanogaster*) indicates that this piRNA targets specifically the *HeT-A* sequence in *D. melanogaster*. Although either *TART* or *TAHRE*, or both, should contribute to *HeT-A* transposition by providing enzymatic activities, their regulation may be somehow less sophisticated. The copy number of *TAHRE* is very low and although many copies of *TART* are present at the telomeres of any given stock [6], a low level of enzymatic activities might be enough to achieve *HeT-A* terminal transposition when needed. In this scenario, a stronger regulation of TART expression would be compatible with the currently most accepted model of a collaborative effort of *HeT-A* and *TART* in telomere maintenance [12]. Alternatively, *TART* might have also acquired a sophisticated strategy to evade regulatory control by piRNAs that still needs to be identified. Moreover, we have also shown that the HeT-A_pi1 piRNA target is conserved in the orthologue copies of the *HeT-A* retrotransposon in three other species of the melanogaster group, thus suggesting that it became fixed nearly 5–10 million years ago. Of course, we can neither exclude some other function for the HeT-A_pi1 region in these other three Drosophila species, nor ensure that HeT-A_pi1 is a piRNA target in them, since no extensive small RNA sequencing has yet been carried out for these species. However, both the extreme nucleotide conservation and the age of this sequence suggest a similar function in all analyzed species.

Our study and others [16], [17] have shown a considerable number of piRNAs matching different target sequences along the *HeT-A* retrotransposon which would be by far sufficient to regulate *HeT-A* transcription. Most intriguingly, only the piRNA target HeT-A_pi1 is conserved across all active copies in *D. melanogaster* and other species of its group. Because the *HeT-A* retrotransposon fulfills an essential function through its active transposition the striking conservation of only this piRNA target sequence also suggests an alternative explanation linked to a functional role in telomere chromatin. Yin and Lin [21] found a piRNA located in the 3R subtelomeric region that binds the Piwi protein and opens the heterochromatin in this genomic region suggesting a crucial role in telomere regulation. Interestingly, in a recent report, Khurana et al. [23] find a direct link between the presence of a subset of piRNAs from the telomeres bound to the Piwi protein and the recruitment of the capping protein complex that protects the telomeres. In those cases one would expect these short sequences and their processing as piRNA be conserved in evolution.

We believe that these two examples together with the striking conservation that we have shown here for the HeT-A_pi1 piRNA target across species, strongly suggests that this could be a case of an alternative or additional functional role other than fine-tuning transcriptional control. The putative dual role of the HeT-A_pi1 piRNA target could have allowed the *HeT-A* retrotransposon to master its role at maintaining Drosophila telomeres. Future work in this direction will hopefully shed enough light to discern the nature of the conservation of this small DNA sequence and the alternative mechanism of Drosophila telomeres.

### Conclusions

The extreme degree of conservation (both within and among species) of the *HeT-A* piRNA target sequence, HeT-A_pi1, particularly in contrast with the high variability of the closely surrounding region, suggests an important function of this sequence in the co-evolution of this TE and the *Drosophila* genome. Two hypotheses are proposed to explain the function of this conserved piRNA target sequence: (1) The fixation of this piRNA target within the sequence of the *HeT-A* retrotransposon could be a truce in the arms race between the telomeric retrotransposon and the fly genome, allowing a highly sophisticated fine-tuned transposition of this particular retrotransposon to the end of the chromosome. (2) The piRNA target HeT-A_pi1 could have an additional and unique function related to telomeric chromatin, protection or function. The latter case would be an example of co-evolution between the *HeT-A* retrotransposon and the Drosophila genome. The new world of recently discovered piRNAs opens a high number of possibilities to study how TEs might have influenced genome evolution.

## Materials and Methods

### Accession Numbers of Used Sequences

The sequences of all *D. melanogaster* complete and canonical transposable elements were obtained from FlyBase [34]. *HeT-A* elements: *HeT-A*{}6262 (FBti0102105), *HeT-A*{}6268 (FBti0102111), *HeT-A*{}6274 (FBti0102117), *HeT-A*{}6265 (FBti0102108), *HeT-A*{}4800 (FBti00062861) and 23Znk (U06920.2). *gypsy1*: M12927.1. *Copia*: X02599.1. *TART*: AY561850.1. *Jockey*: M22874.1. *accord2*: AF541947. I: M14954.2. The reference sequence used for *HeT-A* elements was *HeT-A*{}6268 (FBti0102111). The coordinates of the functional parts of this element are: 5′UTR: 1.914, Gag protein: 915.3749, 3′UTR: 3750.6012. Annotated TE genomic sequences and mRNA sequences were obtained from UCSC database (http://genome.ucsc.edu; tables/repeatMasker and tables/all_mRNAs, respectively). Small RNA reads where obtained from NCBI Sequence Read Archive: SRA007727/SRX002242-5 [17].

### TE Databases

To construct the different Blast databases we used the program makeblastdb from the NCBI/blast2.2.22+ package [35]. For each Drosophila species with genome sequences available, we extracted the sequences of HeT-A elements annotated with RepeatMasker from the UCSC Mysql database (filter “HETA”). The number of HeT-A sequences for each species are presented in Table S1. For species belonging to the melanogaster subgroup we extracted sequences annotated as *TART-B1*, *copia*, *I*, *gypsy1*, *jockey* and *accord2* by RepeatMasker. Different databases were constructed for each element and species. TE sequences annotated by RepeatMasker include both complete and truncated elements.

### Sequence Alignments and Nucleotide Diversity Analyses

All alignments were obtained using Muscle3.6 software [36]. Estimates of nucleotide diversity among elements and species were computed using DNAspv5 [26]. Estimates of nucleotide diversity are obtained from the average number of pairwise nucleotide differences among sequences, irrespective of them coming from different copies of the element. Full methods for conservation analyses can be found in Methods and Results S1.

### Blast Analyses

Blast analyses where performed using local Blast (Blast2.2.22; [35]). To search for small RNA sequences the blastn algorithm was modified to find small sequence hits with at least 24 consecutive identical nucleotides, that is the minimun described length for a piRNA. To find piRNA target sequences matching *HeT-A* copies, each copy was split in overlapping fragments of 30 ntds (with a step of 1 ntds) and the fragments were blasted against the database of small RNA reads database from Li et al. SRA007727/SRX002242-5 [17].

To search for *gag* coding regions, Gag protein sequences were blasted using the tblastn algorithm. The Gag protein in *HeT-A*{}6268 was t-blasted against the database of *HeT-A* annotated sequences from *D. simulans* and *D. sechellia*. In the same way the Gag protein of the *HeT-A* element AF043258, [24] from *D. yakuba* was t-blasted against the database of *HeT-A* annotated sequences from *D. yakuba*.

All reported results of Blast analyses are significant.

### Conservation Analysis of piRNAs in Different Species

Conserved piRNA targets among *D. melanogaster HeT-A* copies (HeT-A_pi1 and HeT-A_pi2) and the 250 ntds sequence where they are contained (starting from 100 ntds before the beginning of each piRNA) were blasted against databases of annotated *HeT-A* sequences in four melanogaster related species (*D. melanogaster*, *D. yakuba*, *D. simulans* and *D. sechellia*). The shared hits between the two blast analyses (blast of piRNAs against TE dbs and blast of sequences of 250 ntds containing the piRNAs against TE dbs) were counted. The proportion of the sequences resulting of the blast of the sequences of 250 ntds without the piRNA was taken as an indicative of the piRNA deletion.

A rough estimation of the conservation of the piRNA for each species was obtained dividing the nucleotide diversity estimates for each piRNA by the nucleotide diversity of the surrounding TE sequence (hits of the Blast analysis of the 250 ntds long sequence) and multiplying this ratio by the proportion of TE sequences containing the piRNA.

To test whether the degree of conservation of piRNAs was significantly different from that of the surrounding region, we devised a likelihood-ratio test. The test is based in comparing the number of observed differences within the piRNA vs. differences in the neighboring 250 ntds region. We use the sequence alignments obtained from the blast analyses to count the relative number of sequence changes harbored by the piRNA and its neighboring 250 ntds region between different copies of the same element within each species. The likelihood of the probabilities of nucleotide changes inside and outside of the piRNA target sequence was estimated under two alternative models (Models 1 and 2). Both models used a binomial distribution where the number of trials is the length of the sequence and the number of successes is the actual number of observed changes. Under Model 1 the piRNA sequence and its neighboring sequences have the same probability of nucleotide changes. Under Model 2 the probability of nucleotide changes is different within the piRNA sequence that in the surrounding region. The basic likelihood function under these two models is as follows:
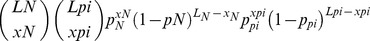
Where *L_pi_* and *L_N_* are, respectively, the lengths in ntds of the piRNA sequence and the neighboring sequence surronding it (a window of 250 ntds was used); *x_pi_* and *x_N_* are the number of nucleotide changes observed in these two sequences and *p_N_* and *p_pi_* are the estimated probability of changes. Under Model 1 *p_N_*  =  *p_pi_* while under Model 2 *p_N_* >*p_pi_*. A log-likelihood ratio test was used to test if Model 2, that contains an extra parameter, is significantly better in explaining our observations than Model 1. The obtained p-values and nucleotide diversity estimates for each species are presented in Table S7. A similar approach was used to find conserved piRNAs in other transposable elements (see Methods and Results S1 and Figure S9).

### RNA Extraction

Ovaries and testicles from adult females and males of *D.* *melanogaster* Oregon R strain were dissected and used to perform RNA extraction (RNeasy® Mini Kit, Qiagen ref.74104). DNAse I treatment as follows: once with RNase-Free DNase set (Qiagen ref.79254) on-column, as manufacturer instructions and twice for 3 hours with the same DNAse I in solution, as manufacturer instructions. The RNA concentration and quality were checked using NanoDrop® ND-1000.

### HeT-A Transcript Amplification and Cloning

Conserved regions from *HeT-A* sequences available in the FlyBase [34] were considered to design primers: 3UTRHeTbF (5′ GCTCCAAGCTGCCAATCC 3′) and *HeT-A* 3′ final reverse (5′ ATTCTGTTCCGCATCCAC 3′), in order to amplify the 3′ UTR region containing the piRNA target HeT-A_pi1 sequence. Amplification was performed by RT-PCR (Transcriptor One-Step RT-PCR Kit (Roche ref. 04655877001) as directed by the manufacturer) specific for sense transcripts amplification. The product of the amplification was directly ligated into pST-Blue 1 plasmid, using the AccepTor™ Vector Kit (Novagene ref. 70595-3). Plasmid DNA was purified by standard alkaline miniprep protocol. Insert presence was checked by EcoRI (Fermentas ref. #ER0271) restriction. The plasmid DNA was sequenced by the Value Service of Macrogen (Korea) using the T7 promoter primer.

## Supporting Information

Figure S1 **Sliding windows showing the nucleotide diversity of the last 400 nucleotides of the** ***HeT-A*** **3′ UTR in different Drosophila species.**
(PDF)

Figure S2 **Position of HeT-A_pi1 relative to the sense and antisense promoters and start sites.** Positions in accordance with the sequence from clone HeT-A{}4R6262 are shown.
(PDF)

Figure S3 **Correlation between the number of piRNAs targeting the complete six** ***HeT-A*** **copies from** ***D.melanogaster*** **and nucleotide diversity among copies.**
(PDF)

Figure S4 **Correlation between the number of piRNAs targeting the complete six** ***HeT-A*** **copies from** ***D.melanogaster*** **and nucleotide diversity among copies without windows conatining the** ***gag*** **coding region sequences.**
(PDF)

Figure S5 **Correlation between the number of piRNAs targeting five** ***I*** **copies from** ***D.melanogaster*** **and nucleotide diversity among copies.**
(PDF)

Figure S6 **Correlation between the number of piRNAs targeting five** ***gypsy1*** **copies from** ***D.melanogaster*** **and nucleotide diversity among copies.**
(PDF)

Figure S7 **Correlation between the number of piRNAs targeting five** ***accord2*** **copies from** ***D.melanogaster*** **and nucleotide diversity among copies.**
(PDF)

Figure S8 **Correlation between the number of piRNAs targeting five** ***copia*** **copies from** ***D.melanogaster*** **and nucleotide diversity among copies.**
(PDF)

Figure S9 **Frequency distribution of RNA reads across six** ***HeT-A*** **copies.**
(PDF)

Figure S10 **Diagram of the approach to find conserved piRNA targets in TEs among copies and species.**
(PDF)

Figure S11 **Conservation index of the fifteen highly conserved piRNA target sequences in the different Drosophila species.**
(PDF)

Figure S12 **Mean values of conservation index (red) and constraints (blue) of the fifteen highly conserved piRNAs target sequences among** ***D. melanogaster, D. simulans, D. sechellia*** **and** ***D. yakuba*** **species.**
(PDF)

Table S1 **Regions significantly conserved among complete** ***HeT-A*** **elements.**
(XLS)

Table S2 **Blast hits along the functional domains of the** ***HeT-A*** **copy HeT-A{}6268.**
(XLS)

Table S3 **Correlation analyses between nucleotide diversity and the number of piRNA target sequences.**
(XLS)

Table S4 **Coordinates of the non-TE mRNAs carrying the HeT-A_pi1 sequence.**
(XLS)

Table S5 **Results of nucleotide diversity and mapping analyses of 3′ UTR transcripts from ovaries and testes.**
(XLS)

Table S6 **Coordinates and number of hits obtained for the 98 conserved piRNA targets.**
(XLS)

Table S7 **Estimates for nucleotide diversity, conservation index and p-values for the sixteen most conserved piRNAs among the four species of the melanogaster group.**
(XLS)

Methods and Results S1 (DOC)
