# Supplementary material for: HeT-A_pi1, a piRNA Target Sequence in the Drosophila Telomeric Retrotransposon HeT-A, Is Extremely Conserved across Copies and Species
Source: PLoS One. 2012 May 21;7(5):e37405. doi: 10.1371/journal.pone.0037405 (PMC3357415; doi:10.1371/journal.pone.0037405)
Supplement: Figure S1 — Sliding windows showing the nucleotide diversity of the last 400 nucleotides of the HeT-A 3′ UTR in different Drosophila species. (PDF) [file pone.0037405.s001.pdf]

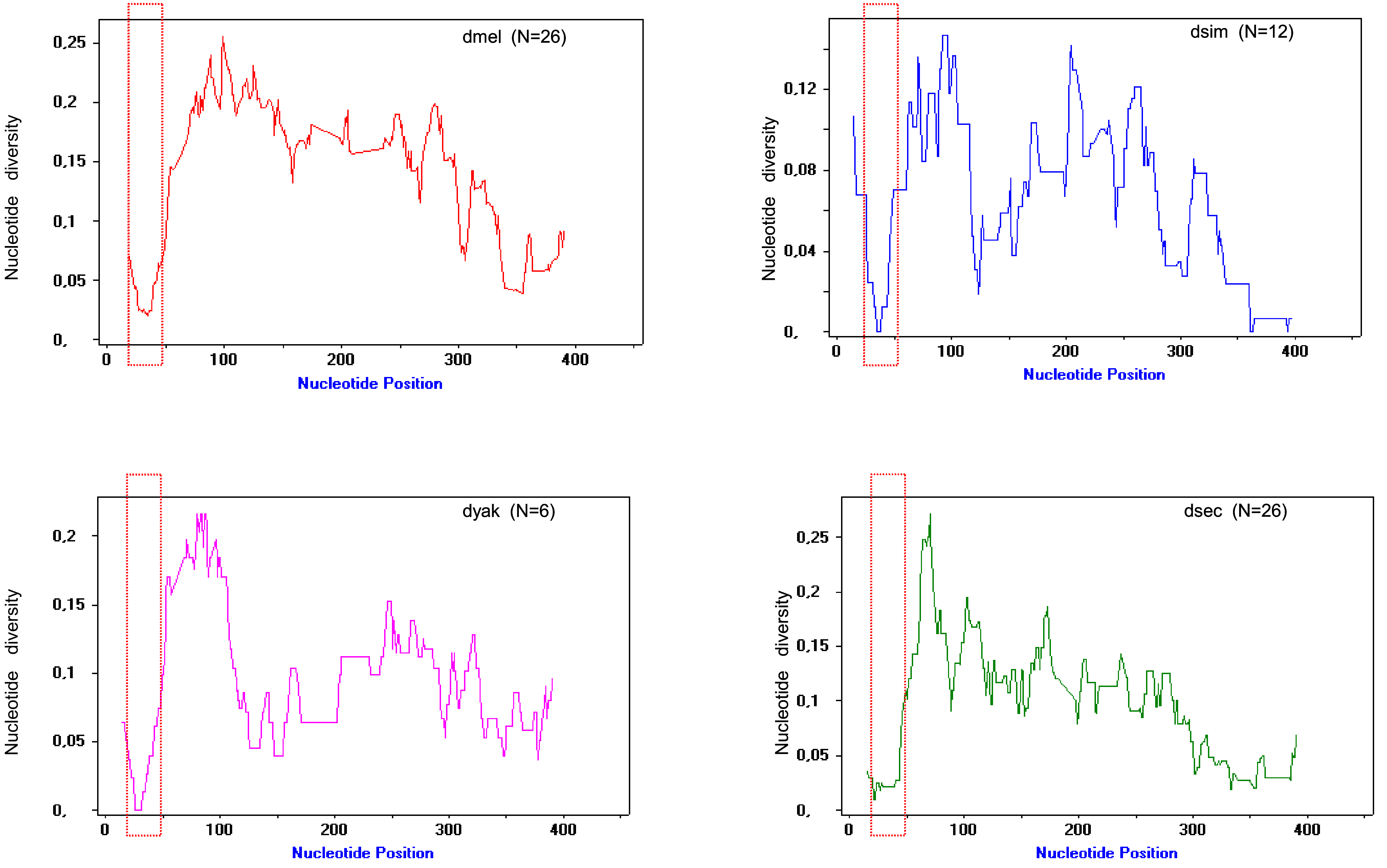

Figure S1. Conservation of the 3' UTR sequence of *HeT-A* in different Drosophila species. Sliding windows (window size=25 ntds, step=1ntd) showing the nucleotide diversity of the last 400 nucleotides of the *HeT-A* 3' UTR in different Drosophila species. The R2 region is labeled with a red rectangle and corresponds to the piRNA target HeT-A_pi1. Only sequences with homology within the last 500bp of the 3'UTR of the HeT-A 4R6268 copy (*D. melanogaster*) and longer than 350 ntds. were used (see supp. Table S2).
