# Supplementary material for: HeT-A_pi1, a piRNA Target Sequence in the Drosophila Telomeric Retrotransposon HeT-A, Is Extremely Conserved across Copies and Species
Source: PLoS One. 2012 May 21;7(5):e37405. doi: 10.1371/journal.pone.0037405 (PMC3357415; doi:10.1371/journal.pone.0037405)
Supplement: Figure S2 — Position of HeT-A_pi1 relative to the sense and antisense promoters and start sites. Positions in accordance with the sequence from clone HeT-A{}4R6262 are shown. (PDF) [file pone.0037405.s002.pdf]

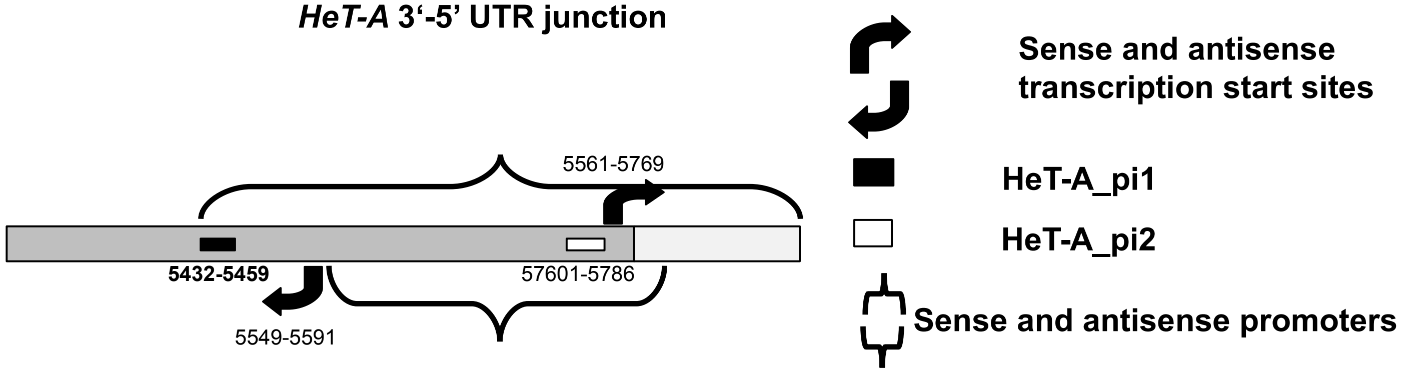

Figure S2. **Position of HeT-A_pi1 relative to the sense and antisense promoters and start sites**. Positions in accordance with the sequence from clone HeT-A{}4R6262 are shown.
