# Supplementary material for: HeT-A_pi1, a piRNA Target Sequence in the Drosophila Telomeric Retrotransposon HeT-A, Is Extremely Conserved across Copies and Species
Source: PLoS One. 2012 May 21;7(5):e37405. doi: 10.1371/journal.pone.0037405 (PMC3357415; doi:10.1371/journal.pone.0037405)
Supplement: Figure S3 — Correlation between the number of piRNAs targeting the complete six HeT-A copies from D.melanogaster and nucleotide diversity among copies. (PDF) [file pone.0037405.s003.pdf]

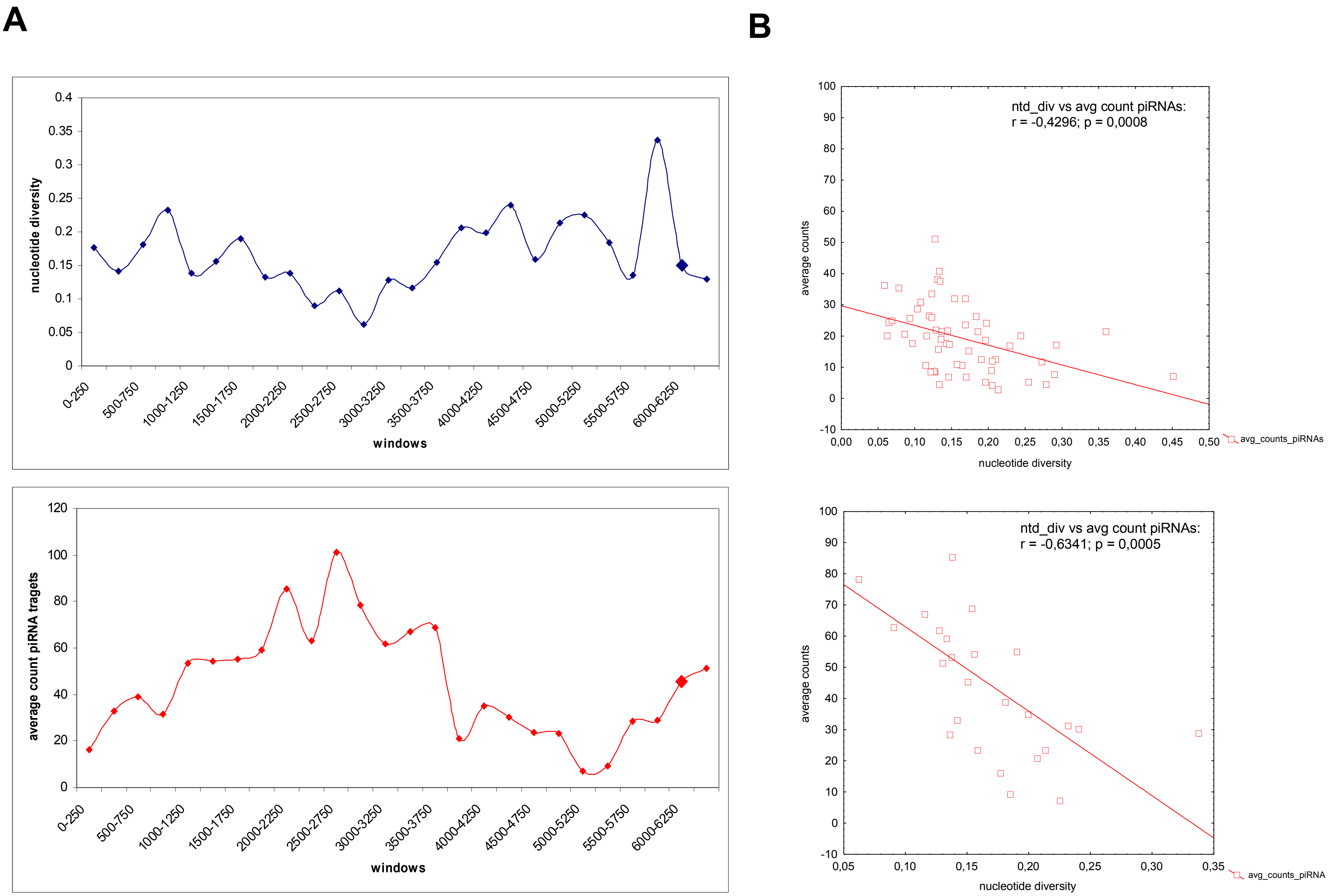

Figure S3. Correlation between the number of piRNAs targeting the complete six *HeT-A* copies from *D.melanogaster* and nucleotide diversity among copies in non overlapping windows of 100 and 250 ntds. **A)** Above, nucleotide diversity along the sequence of the six complete *HeT-A* copies estimated in non-overlapping windows of 250 ntds. Below, average number of piRNAs targeting the *HeT-A* sequence by windows. **B)** Scatterplot of the correlation between the average counts of target piRNAs and the nucleotide diversity among the six complete copies in non overlapping windows of 100 (Above) and 250 (below) nucleotides. Higher diamonds indicate the window where HeT-A_pi1 is.
