# Supplementary material for: HeT-A_pi1, a piRNA Target Sequence in the Drosophila Telomeric Retrotransposon HeT-A, Is Extremely Conserved across Copies and Species
Source: PLoS One. 2012 May 21;7(5):e37405. doi: 10.1371/journal.pone.0037405 (PMC3357415; doi:10.1371/journal.pone.0037405)
Supplement: Figure S4 — Correlation between the number of piRNAs targeting the complete six HeT-A copies from D.melanogaster and nucleotide diversity among copies without windows conatining the gag coding region sequences. (PDF) [file pone.0037405.s004.pdf]

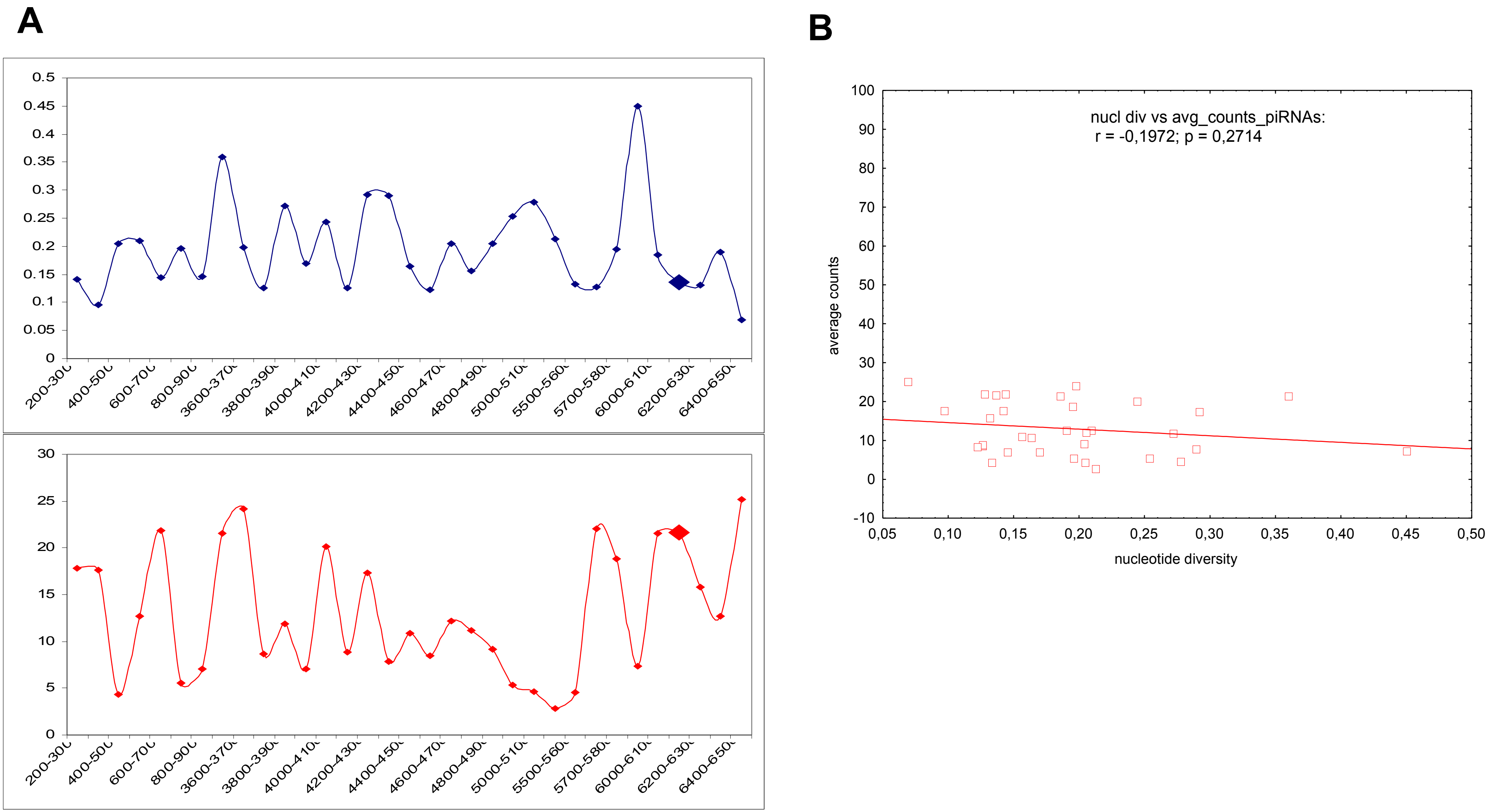

Figure S4: Correlation between the number of piRNAs targeting the complete six *HeT-A* copies from *D.melanogaster* and nucleotide diversity among copies in non overlapping windows of 100 ntds without windows containing gag gene sequences. A) Above, nucleotide diversity along the sequence of the six complete *HeT-A* copies estimated in non-overlapping windows. Below, average number of piRNAs targeting the *HeT-A* sequence by windows. **B)** Scatterplot of the correlation between the average counts of RNA reads and target piRNAs and the nucleotide diversity among the six complete copies in non overlapping windows of 100 nucleotides. Higher diamonds indicate the window where HeT-A_pi1 is.
