# Supplementary material for: HeT-A_pi1, a piRNA Target Sequence in the Drosophila Telomeric Retrotransposon HeT-A, Is Extremely Conserved across Copies and Species
Source: PLoS One. 2012 May 21;7(5):e37405. doi: 10.1371/journal.pone.0037405 (PMC3357415; doi:10.1371/journal.pone.0037405)
Supplement: Figure S5 — Correlation between the number of piRNAs targeting five I copies from D.melanogaster and nucleotide diversity among copies. (PDF) [file pone.0037405.s005.pdf]

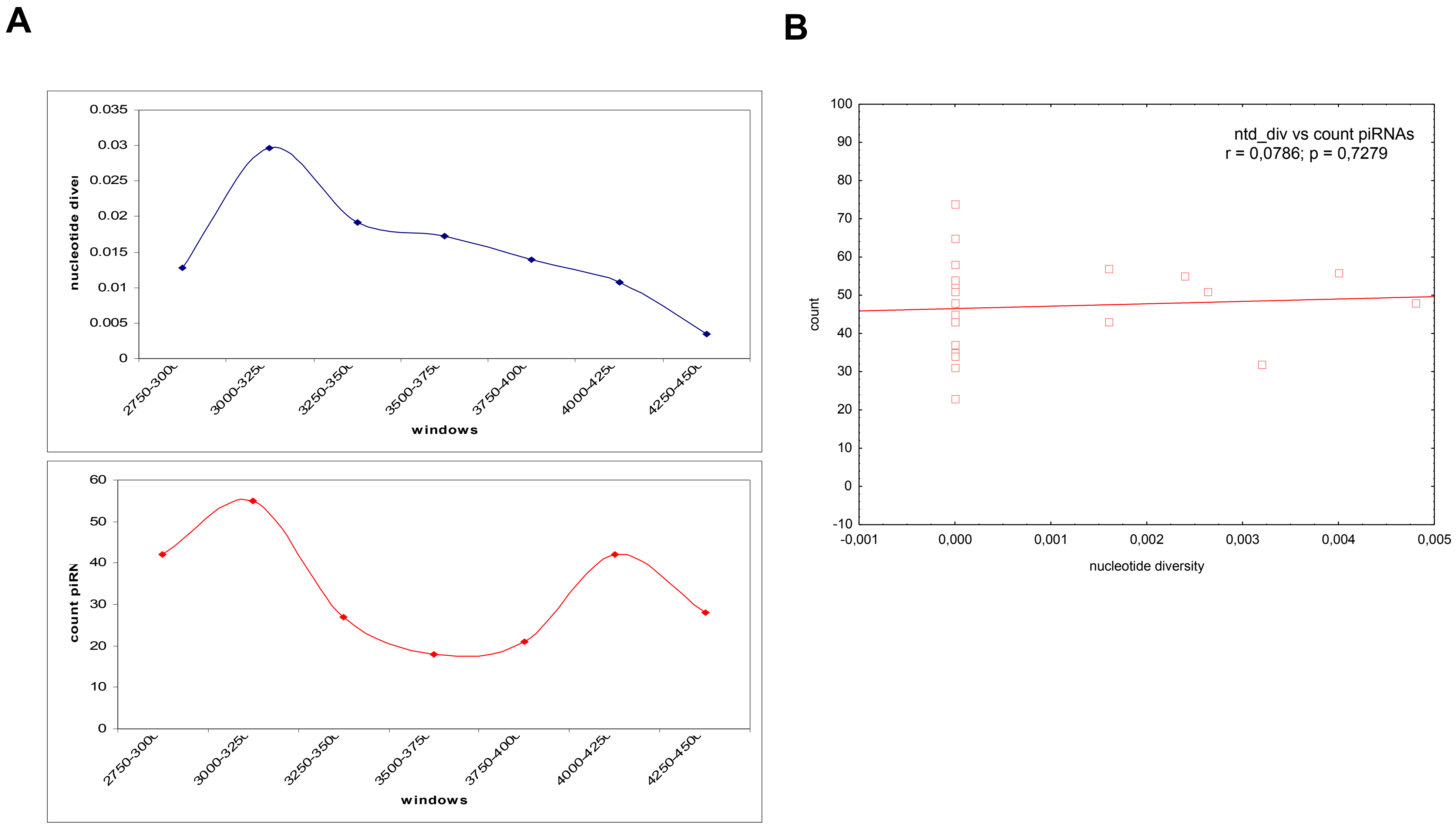

Figure S5: Correlation between the number of piRNAs targeting five *I* retroelement copies from *D. melanogaster* and nucleotide diversity among copies in non overlapping windows of 250 ntds. A) Above, nucleotide diversity along the sequence of five *I* copies estimated in non-overlapping windows. Below, average number of piRNAs targeting the *I retroelement* sequence by windows. **B)** Scatterplot of the correlation between the average counts of target piRNAs and the nucleotide diversity among five copies of *I* retrolement in non overlapping windows of 250 nucleotides.
