# Supplementary material for: HeT-A_pi1, a piRNA Target Sequence in the Drosophila Telomeric Retrotransposon HeT-A, Is Extremely Conserved across Copies and Species
Source: PLoS One. 2012 May 21;7(5):e37405. doi: 10.1371/journal.pone.0037405 (PMC3357415; doi:10.1371/journal.pone.0037405)
Supplement: Figure S7 — Correlation between the number of piRNAs targeting five accord2 copies from D.melanogaster and nucleotide diversity among copies. (PDF) [file pone.0037405.s007.pdf]

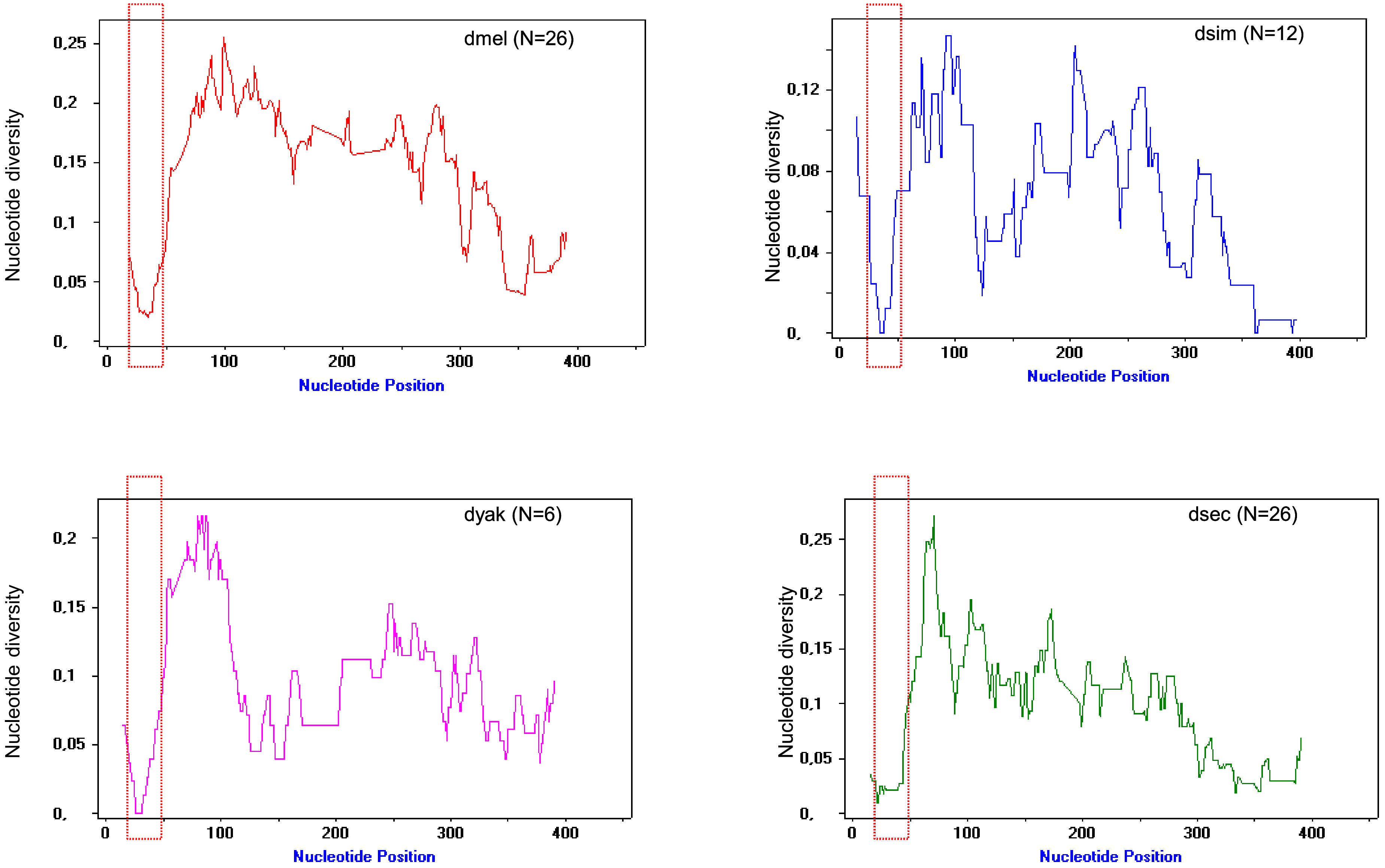

Figure S1. **Conservation of the 3' UTR sequence of *HeT-A* in different Drosophila species**. Sliding windows (window size=25 ntds, step=1ntd) showing the nucleotide diversity of the last 400 nucleotides of the *HeT-A* 3' UTR in different Drosophila species. The R2 region is labeled with a red rectangle and corresponds to the piRNA target HeT-A_pi1. Only sequences with homology within the last 500bp of the 3'UTR of the HeT-A 4R6268 copy (*D. melanogaster*) and longer than 350 ntds. were used (see supp. Table S2).

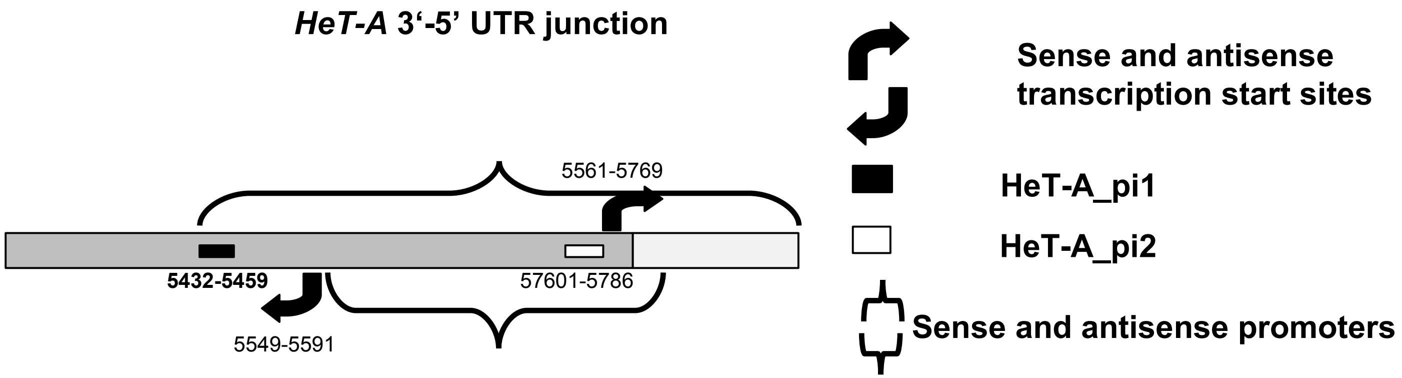

Figure S2. **Position of HeT-A_pi1 relative to the sense and antisense promoters and start sites**. Positions in accordance with the sequence from clone HeT-A{}4R6262 are shown.

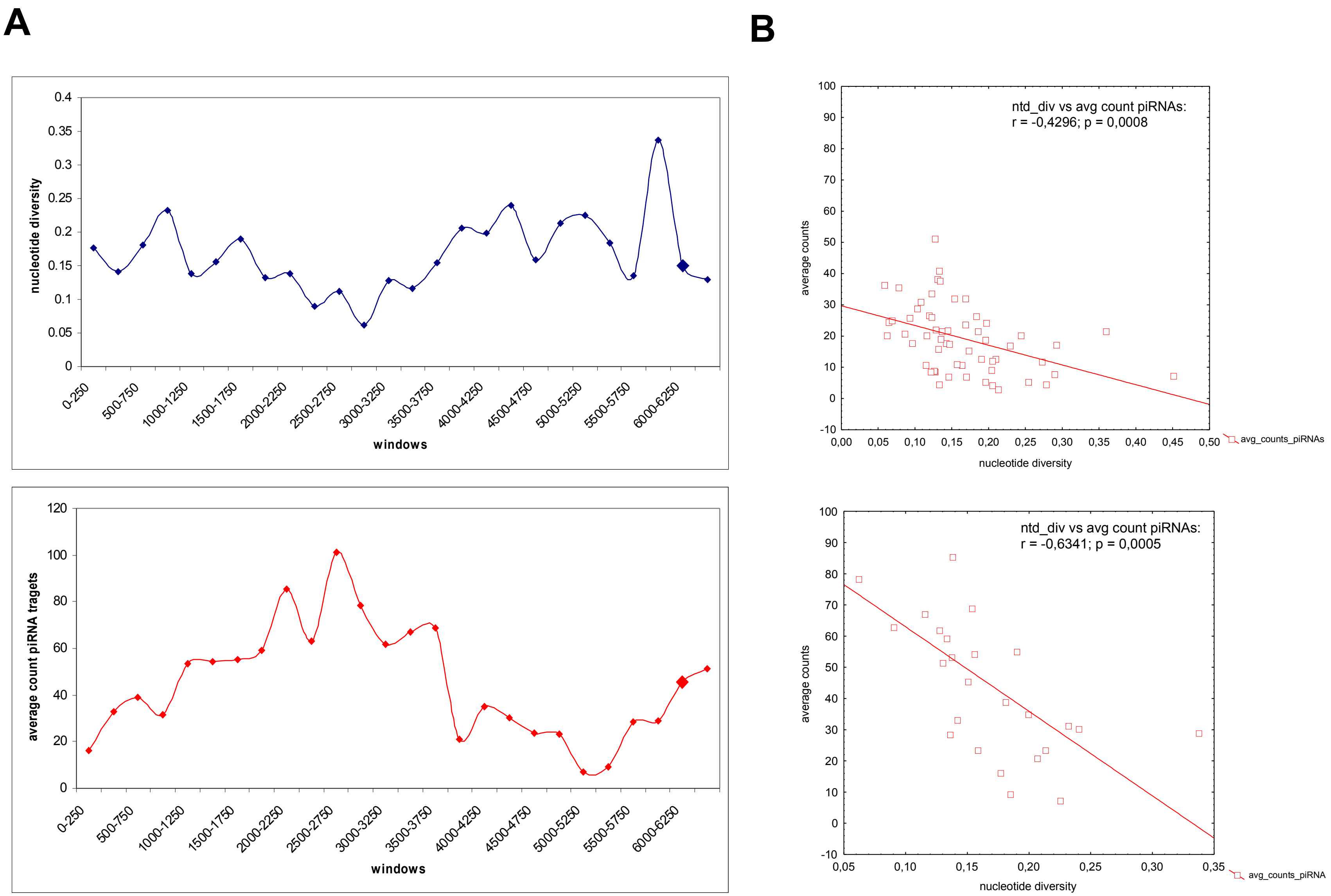

Figure S3. Correlation between the number of piRNAs targeting the complete six *HeT-A* copies from *D.melanogaster* and nucleotide diversity among copies in non overlapping windows of 100 and 250 ntds. A) Above, nucleotide diversity along the sequence of the six complete *HeT-A* copies estimated in non-overlapping windows of 250 ntds. Below, average number of piRNAs targeting the *HeT-A* sequence by windows**. B)** Scatterplot of the correlation between the average counts of target piRNAs and the nucleotide diversity among the six complete copies in non overlapping windows of 100 (Above) and 250 (below) nucleotides. Higher diamonds indicate the window where HeT-A_pi1 is.

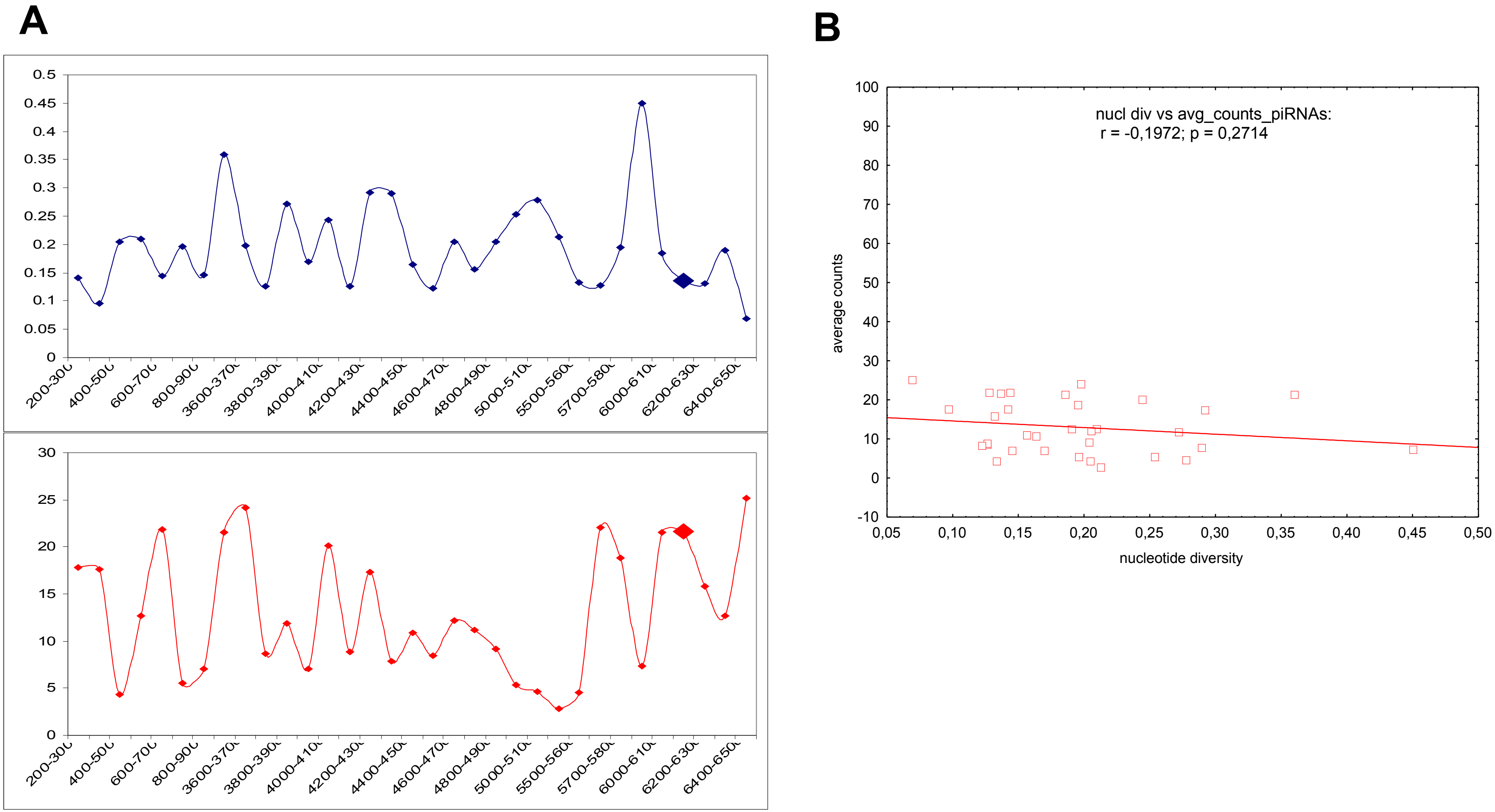

Figure S4: Correlation between the number of piRNAs targeting the complete six *HeT-A* copies from *D.melanogaster* and nucleotide diversity among copies in non overlapping windows of 100 ntds without windows containing gag gene sequences. A) Above, nucleotide diversity along the sequence of the six complete *HeT-A* copies estimated in non-overlapping windows. Below, average number of piRNAs targeting the *HeT-A* sequence by windows. **B)** Scatterplot of the correlation between the average counts of RNA reads and target piRNAs and the nucleotide diversity among the six complete copies in non overlapping windows of 100 nucleotides. Higher diamonds indicate the window where HeT-A_pi1 is.

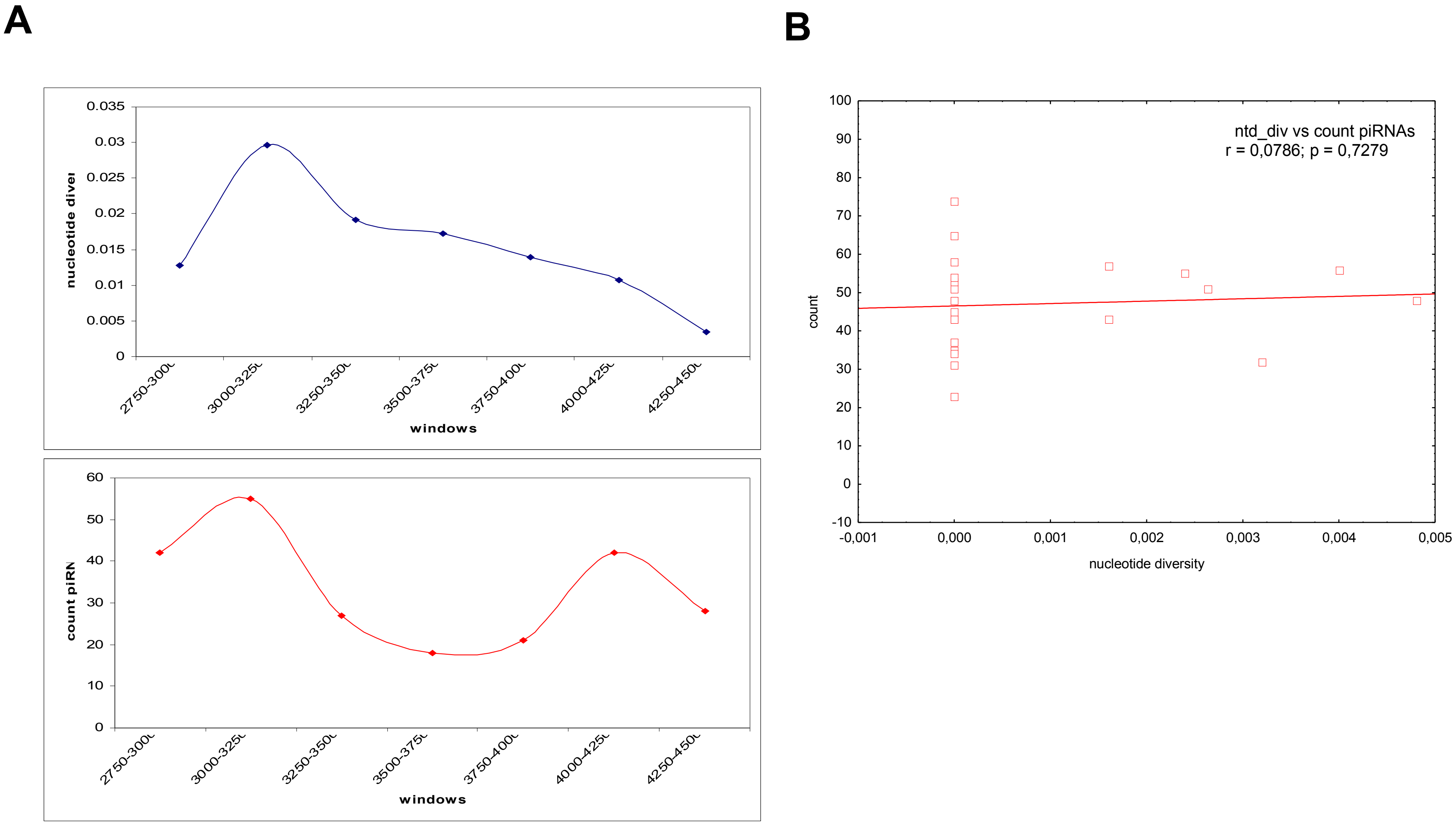

Figure S5: Correlation between the number of piRNAs targeting five *I* retroelement copies from *D. melanogaster* and nucleotide diversity among copies in non overlapping windows of 250 ntds. A) Above, nucleotide diversity along the sequence of five *I* copies estimated in non-overlapping windows. Below, average number of piRNAs targeting the *I retroelement* sequence by windows. **B)** Scatterplot of the correlation between the average counts of target piRNAs and the nucleotide diversity among five copies of *I* retrolement in non overlapping windows of 250 nucleotides.

A

B

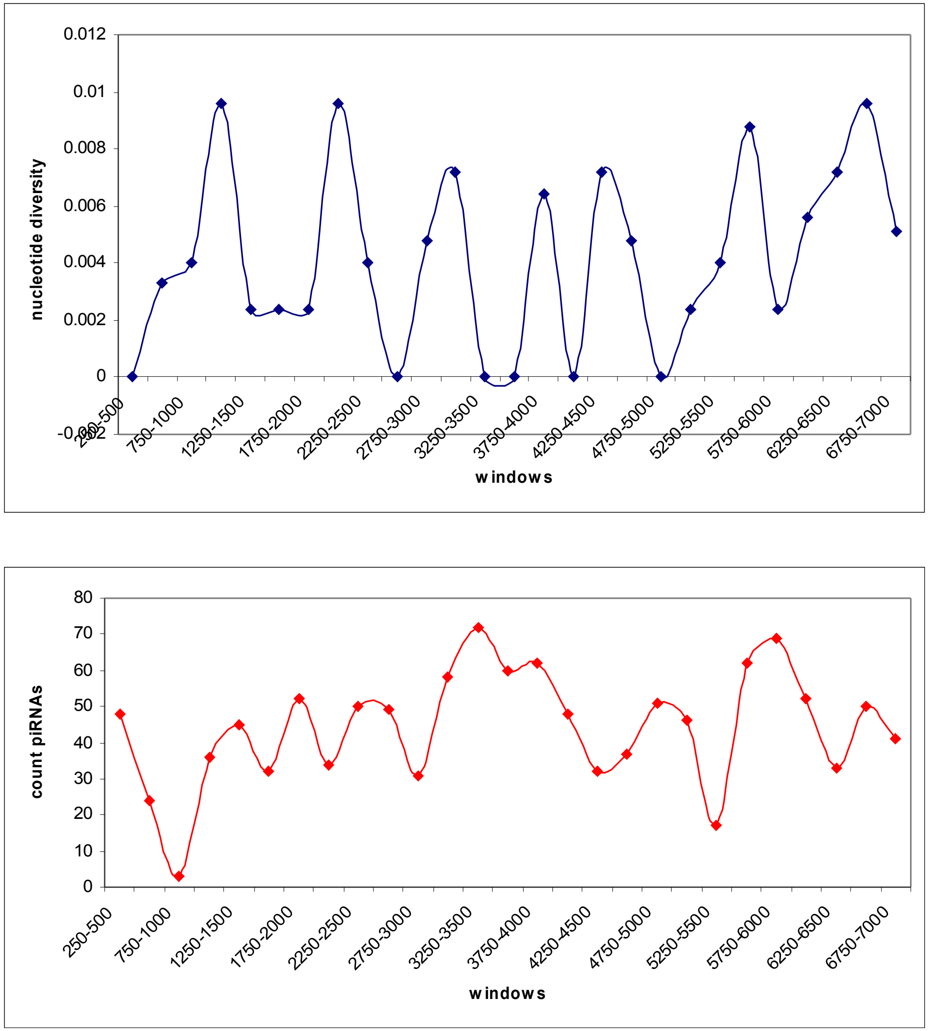

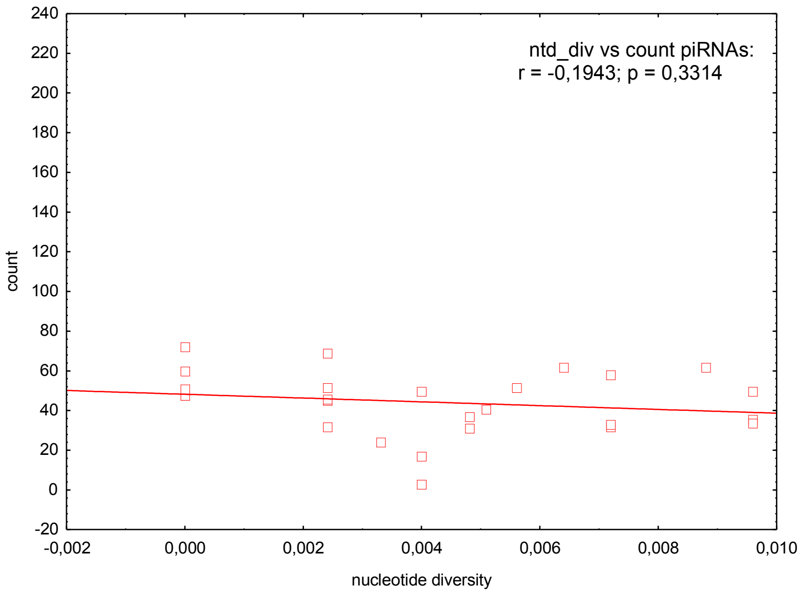

Figure S6: Correlation between the number of piRNAs targeting five *gypsy1* retroelement copies from *D. melanogaster* and nucleotide diversity among copies in non overlapping windows of 250 ntds. A) Above, nucleotide diversity along the sequence of the five *gypsy1* copies estimated in non-overlapping windows. Below, average number of piRNAs targeting the *gypsy1* sequence by windows. **B)** Scatterplot of the correlation between the average counts of target piRNAs and the nucleotide diversity among five copies of *gypsy1* retrolement in non overlapping windows of 250 nucleotides.

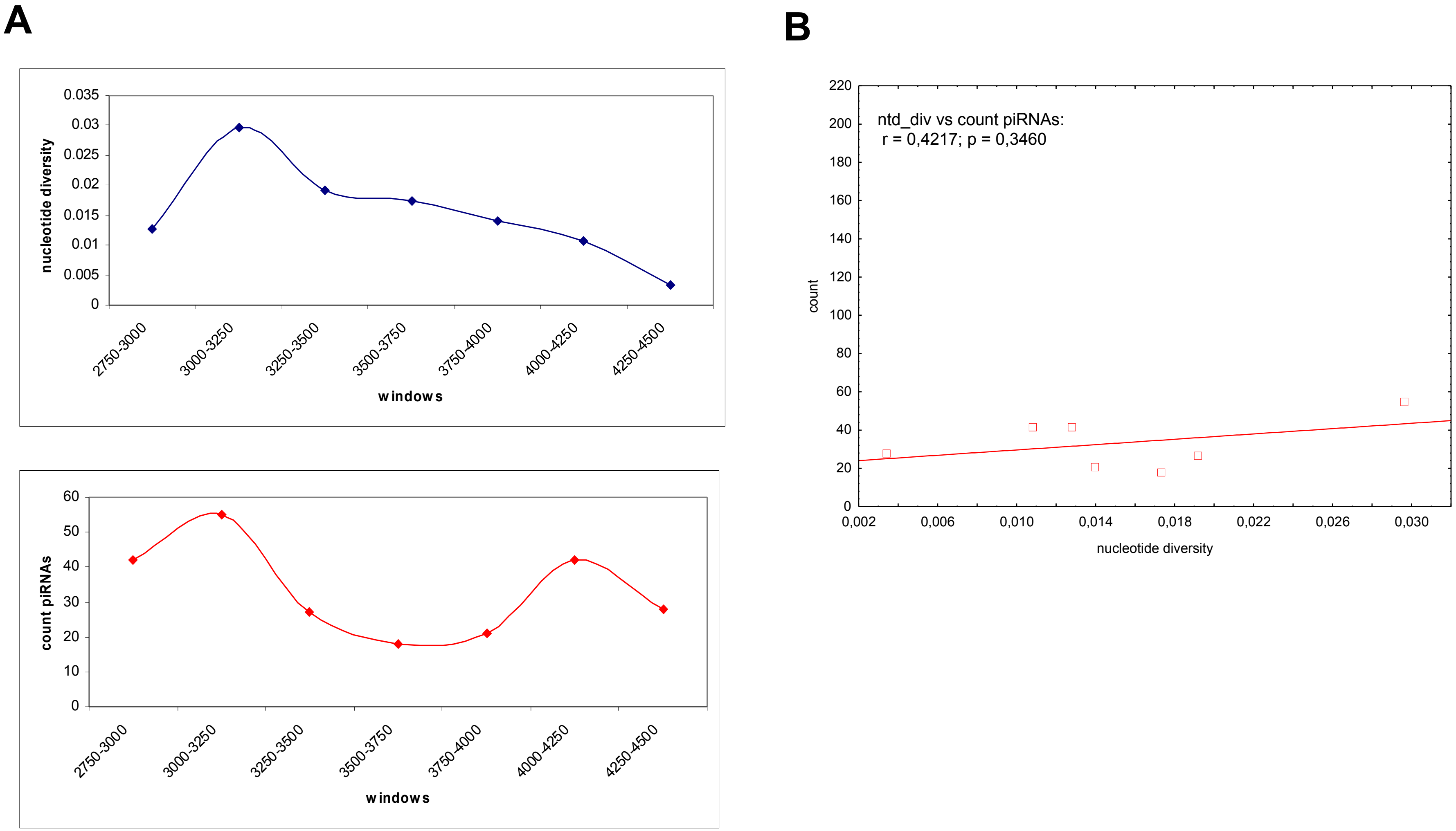

Figure S7: Correlation between the number of piRNAs targeting five *accord2* retroelement copies from *D. melanogaster* and nucleotide diversity among copies in non overlapping windows of 250 ntds. A) Above, nucleotide diversity along the sequence of five *accord2* copies estimated in non-overlapping windows. Below, average number of piRNAs targeting the *accord2* sequence by windows. **B)** Scatterplot of the correlation between the average counts of target piRNAs and the nucleotide diversity among five copies of *accord2* retrolement in non overlapping windows of 250 nucleotides.

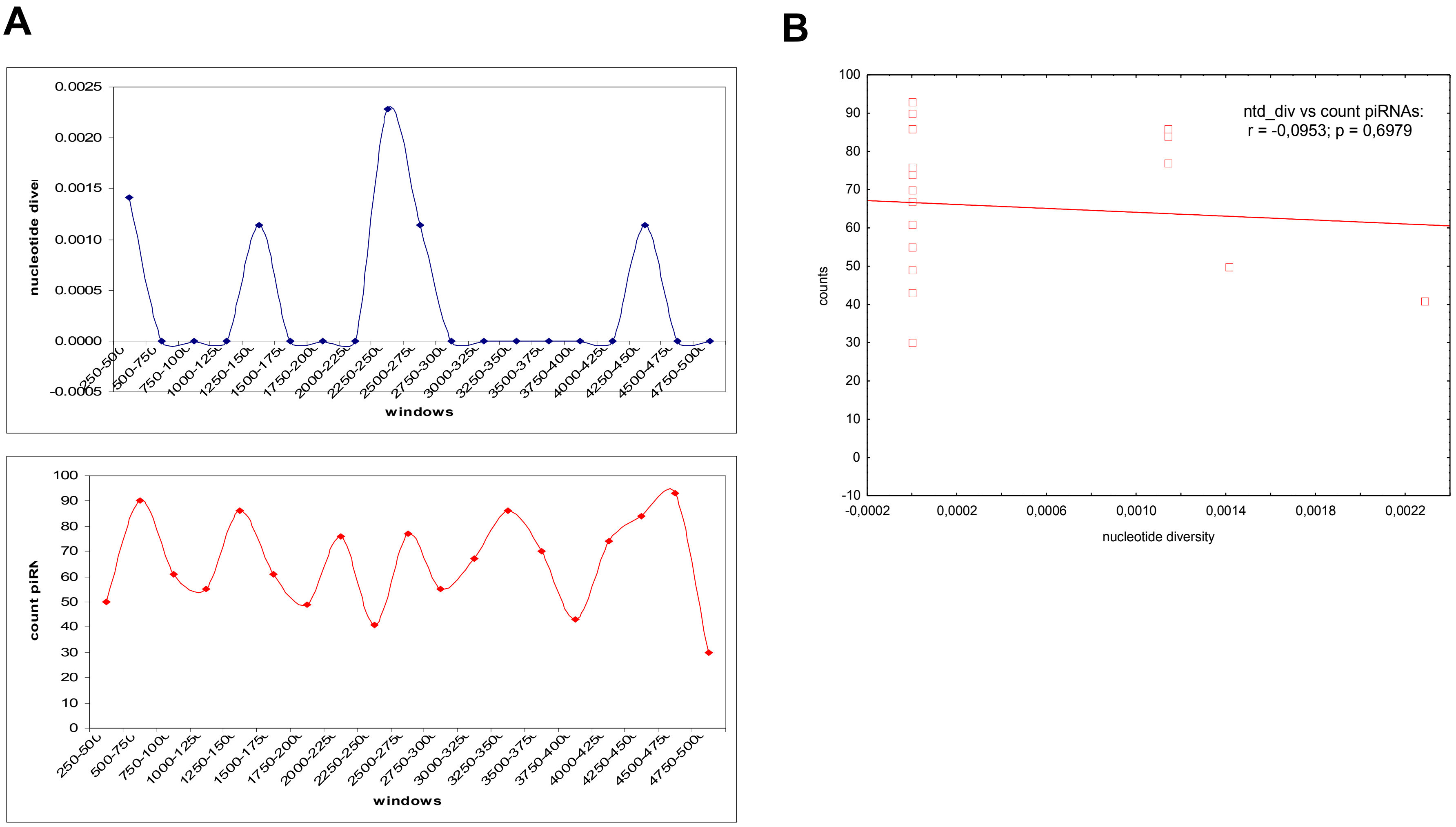

Figure S8: Correlation between the number of piRNAs targeting five *copia* retroelement copies from *D. melanogaster* and nucleotide diversity among copies in non overlapping windows of 250 ntds. A) Above, nucleotide diversity along the sequence of five *copia* copies estimated in non-overlapping windows. Below, average number of piRNAs targeting the *copia* sequence by windows. **B)** Scatterplot of the correlation between the average counts of target piRNAs and the nucleotide diversity among five copies of *copia* retrolement in non overlapping windows of 250 nucleotides.

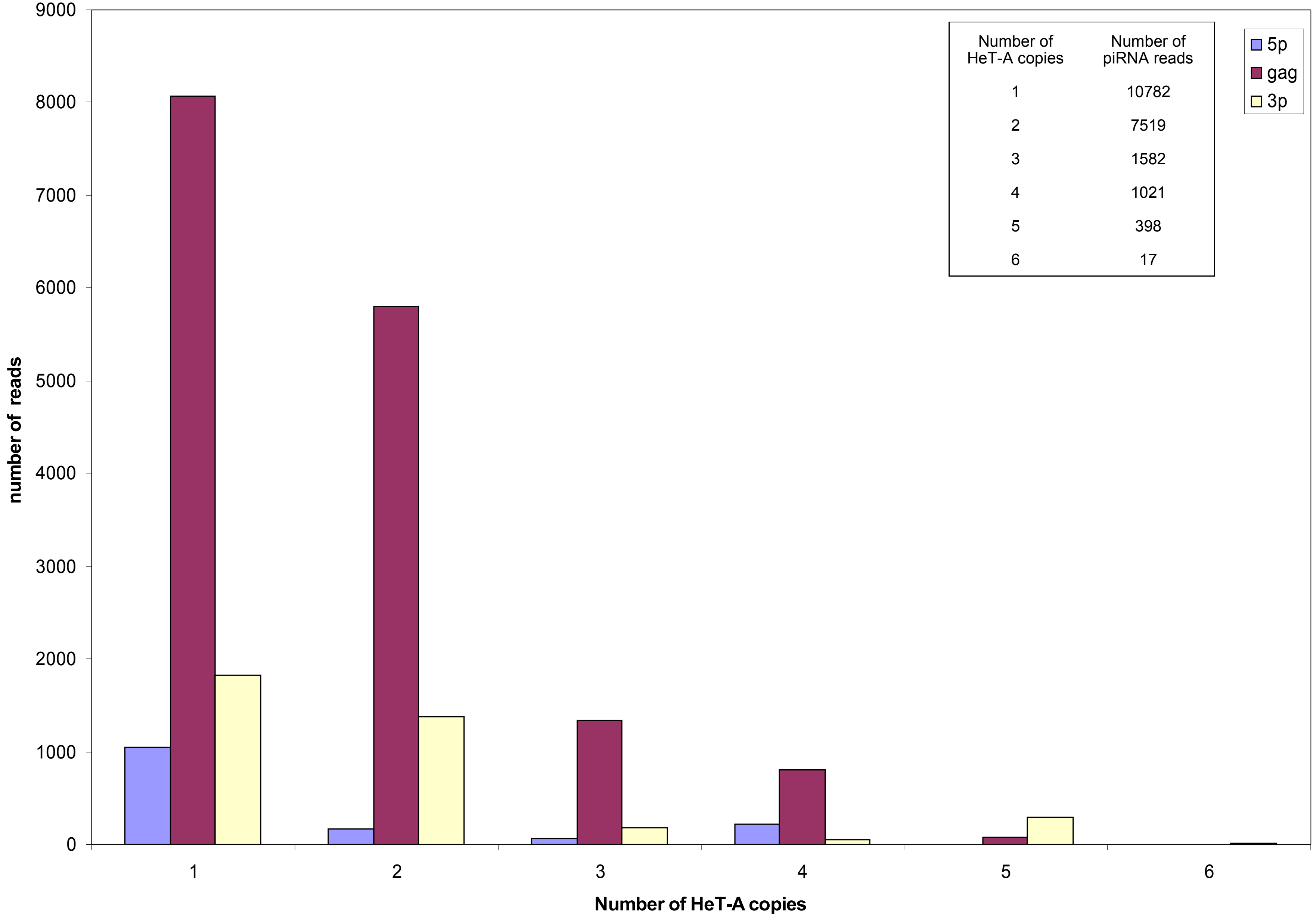

Figure S9. Frequency distribution of the number of piRNA reads targeting *HeT-A* among the six copies from *D. melanogaster*. Different functional parts of the element are market with different colors; blue:5' UTR, yellow: 3' UTR, red: gag coding region. Most of the piRNA reads match one single HeT-A copy, and only 17 reads match the six HeT-A copies. Numbers in the box are the total numbers of piRNA reads matching one copy, two copies and so on.

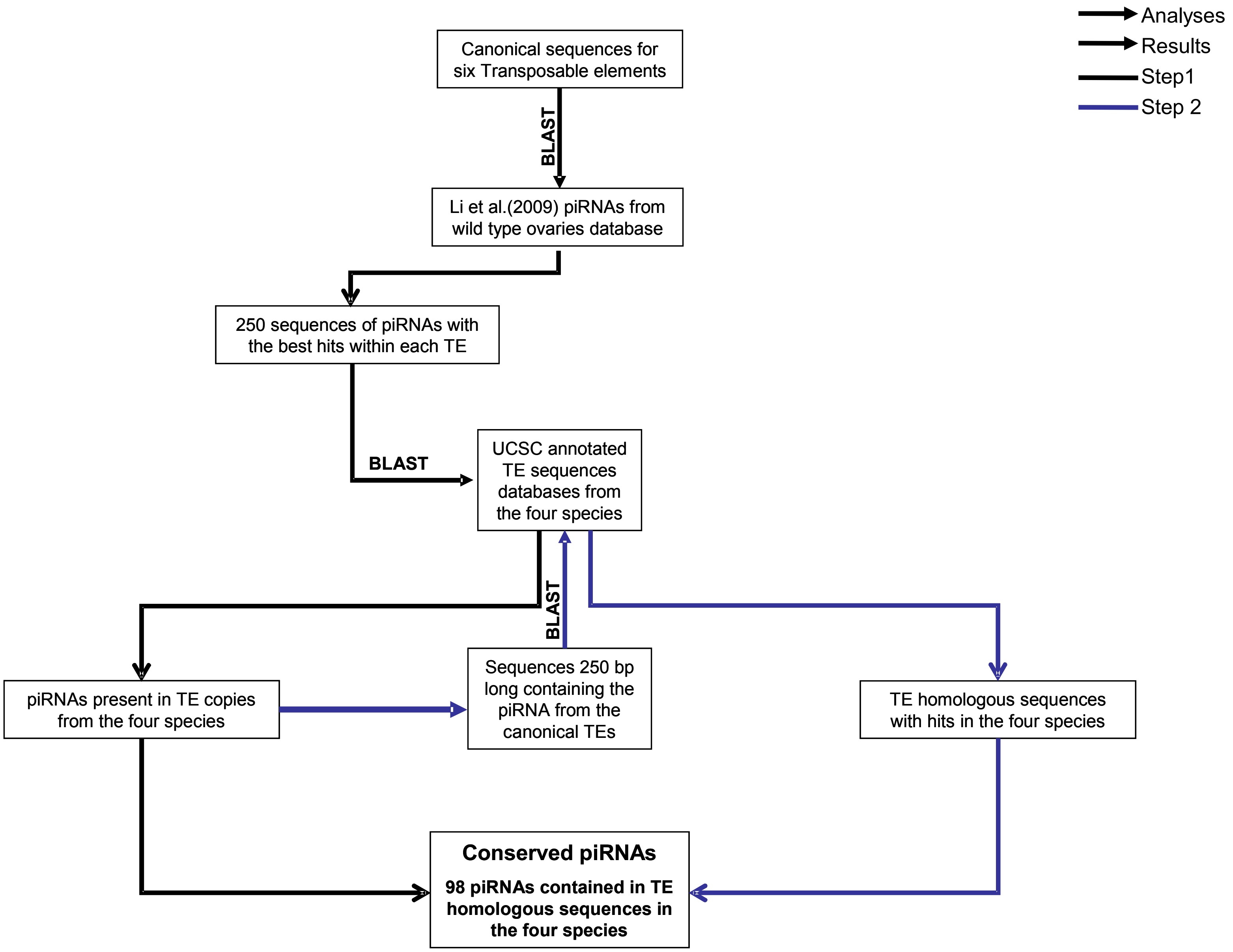

Figure S10. Diagram of the approach to find conserved piRNA targets in TEs among copies and species. Different color arrows indicate two different and alternative steps. Step 1 (black arrows) piRNA target sequences from the database of each annotated TE in all four species were detected. Step 2 (blue arrows) homologous TE sequences containing the piRNA target sequence were obtained. Conserved piRNAs are that present in all four species and within a homologous canonical TE sequence.

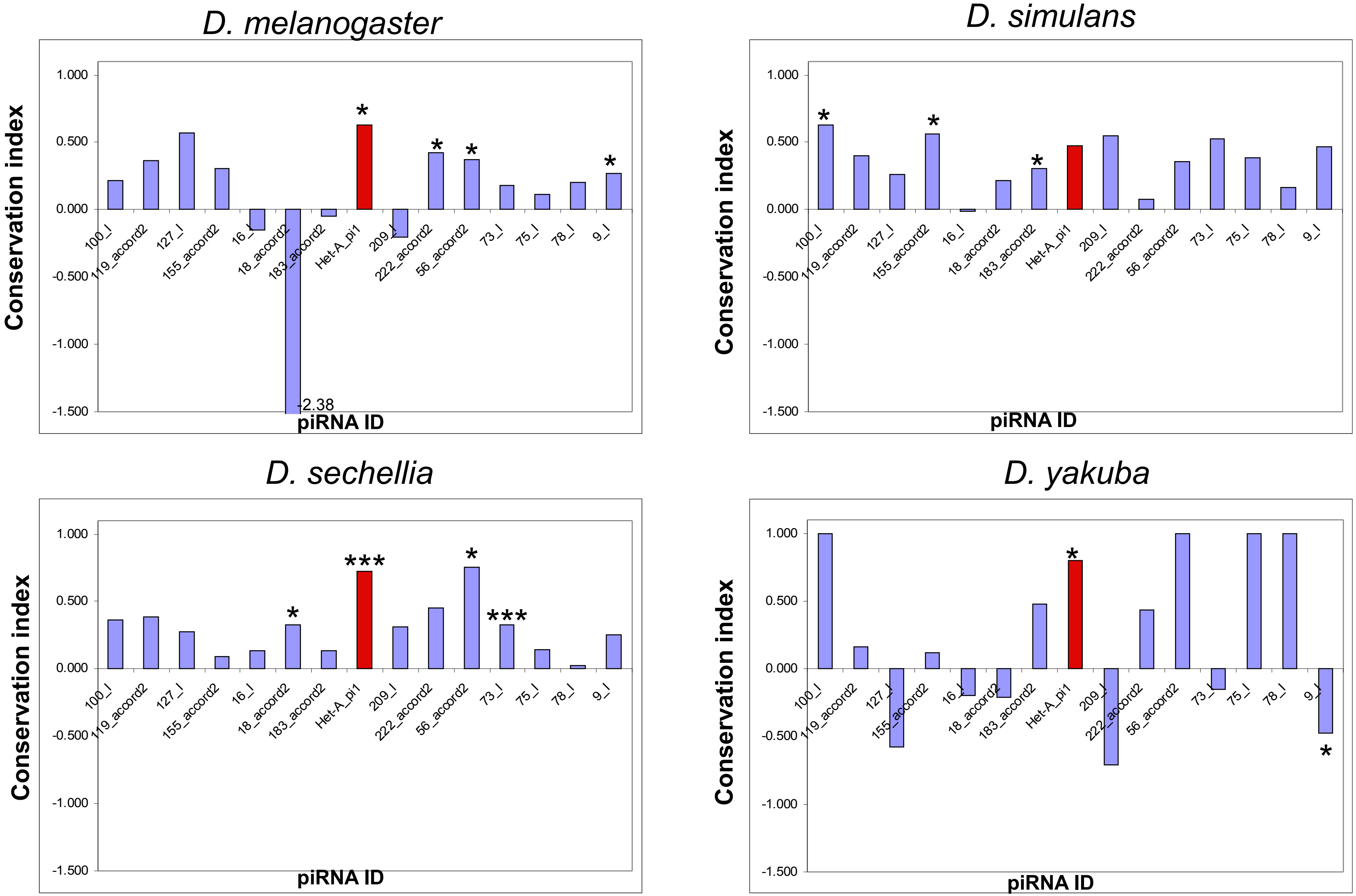

Figure S11. Conservation index of the fifteen highly conserved piRNA target sequences in the indicated species. The HeT-A_pi1 target labeled in red. Positives and negative bars indicate higher or lesser conservation of the piRNA target sequence in respect to the TE sequence where the target is embedded. Stars indicate cases where the number of nucleotide changes of the piRNA target sequence is significantly different than the TE sequence where the target is embedded (*=p<0.05; ***=p<0.005)

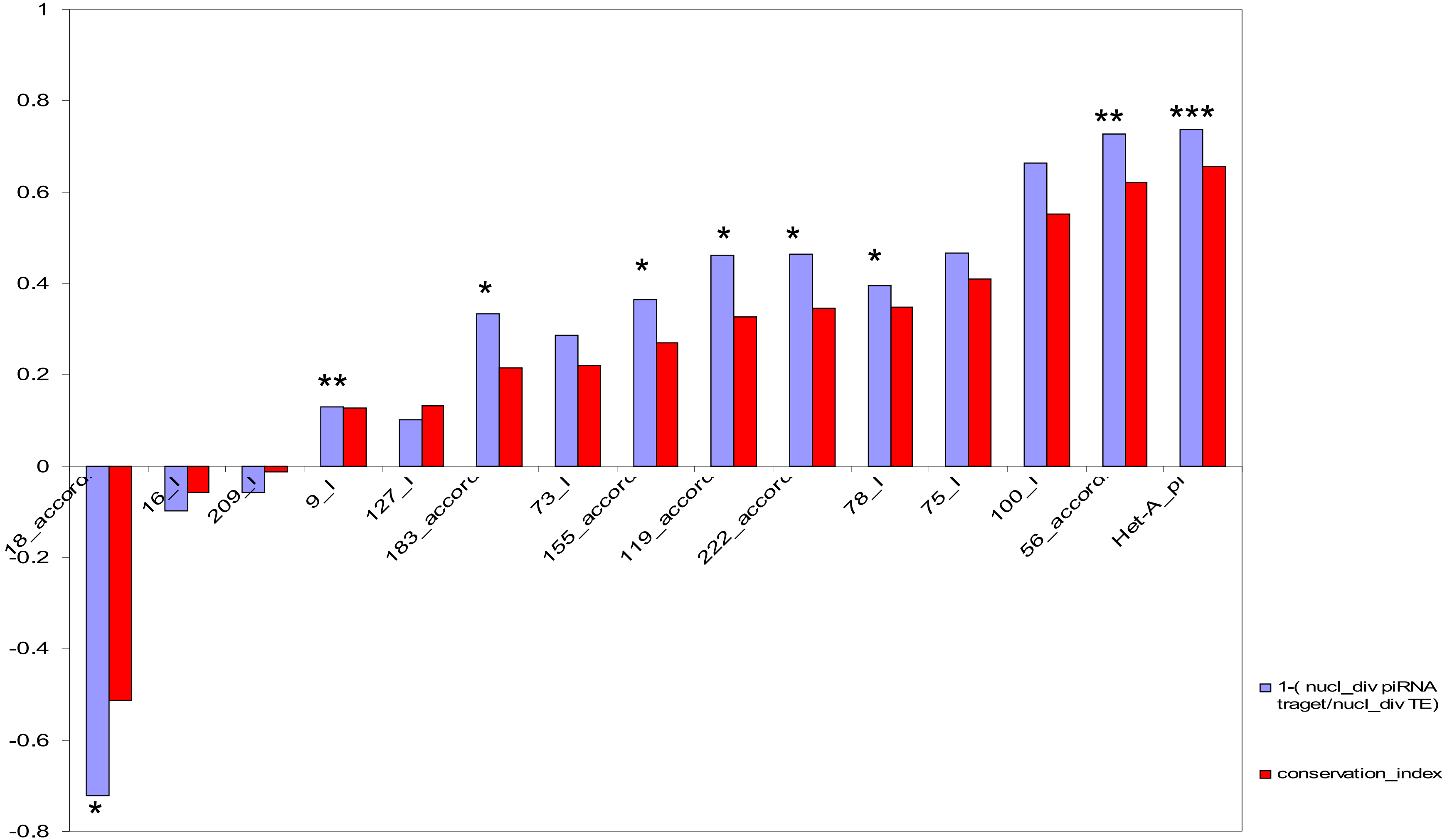

Number of species with signifficantly different number of nucleotide changes between the piRNA and TE sequences

*=1sps; **=2 sps, ***=3 sps

Figure S12. Mean values of conservation index (red) and constraints (blue) of the fifteen highly conserved piRNAs target sequences among *D. melanogaster, D. simulans, D. sechellia* and *D. yakuba* species. Asterisks label those cases (species) where the piRNA target sequence has significantly different number of nucleotide changes than the flanking sequence:
