# Supplementary material for: HeT-A_pi1, a piRNA Target Sequence in the Drosophila Telomeric Retrotransposon HeT-A, Is Extremely Conserved across Copies and Species
Source: PLoS One. 2012 May 21;7(5):e37405. doi: 10.1371/journal.pone.0037405 (PMC3357415; doi:10.1371/journal.pone.0037405)
Supplement: Figure S9 — Frequency distribution of RNA reads across six HeT-A copies. (PDF) [file pone.0037405.s009.pdf]

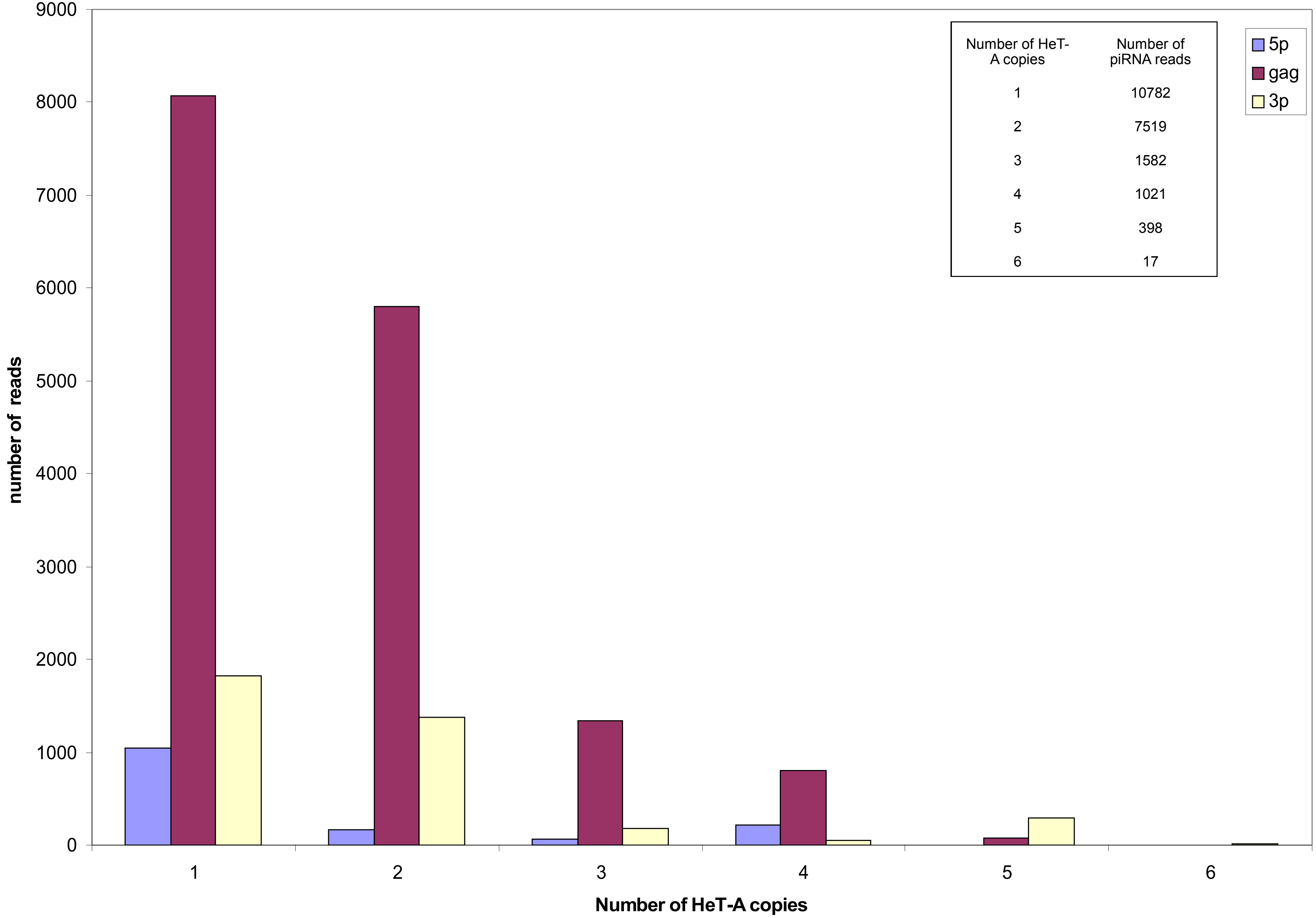

Figure S9. Frequency distribution of the number of piRNA reads targeting *HeT-A* among the six copies from *D. melanogaster*. Different functional parts of the element are market with different colors; blue:5' UTR, yellow: 3' UTR, red: gag coding region. Most of the piRNA reads match one single HeT-A copy, and only 17 reads match the six HeT-A copies. Numbers in the box are the total numbers of piRNA reads matching one copy, two copies and so on.
