# Supplementary material for: HeT-A_pi1, a piRNA Target Sequence in the Drosophila Telomeric Retrotransposon HeT-A, Is Extremely Conserved across Copies and Species
Source: PLoS One. 2012 May 21;7(5):e37405. doi: 10.1371/journal.pone.0037405 (PMC3357415; doi:10.1371/journal.pone.0037405)
Supplement: Figure S10 — Diagram of the approach to find conserved piRNA targets in TEs among copies and species. (PDF) [file pone.0037405.s010.pdf]

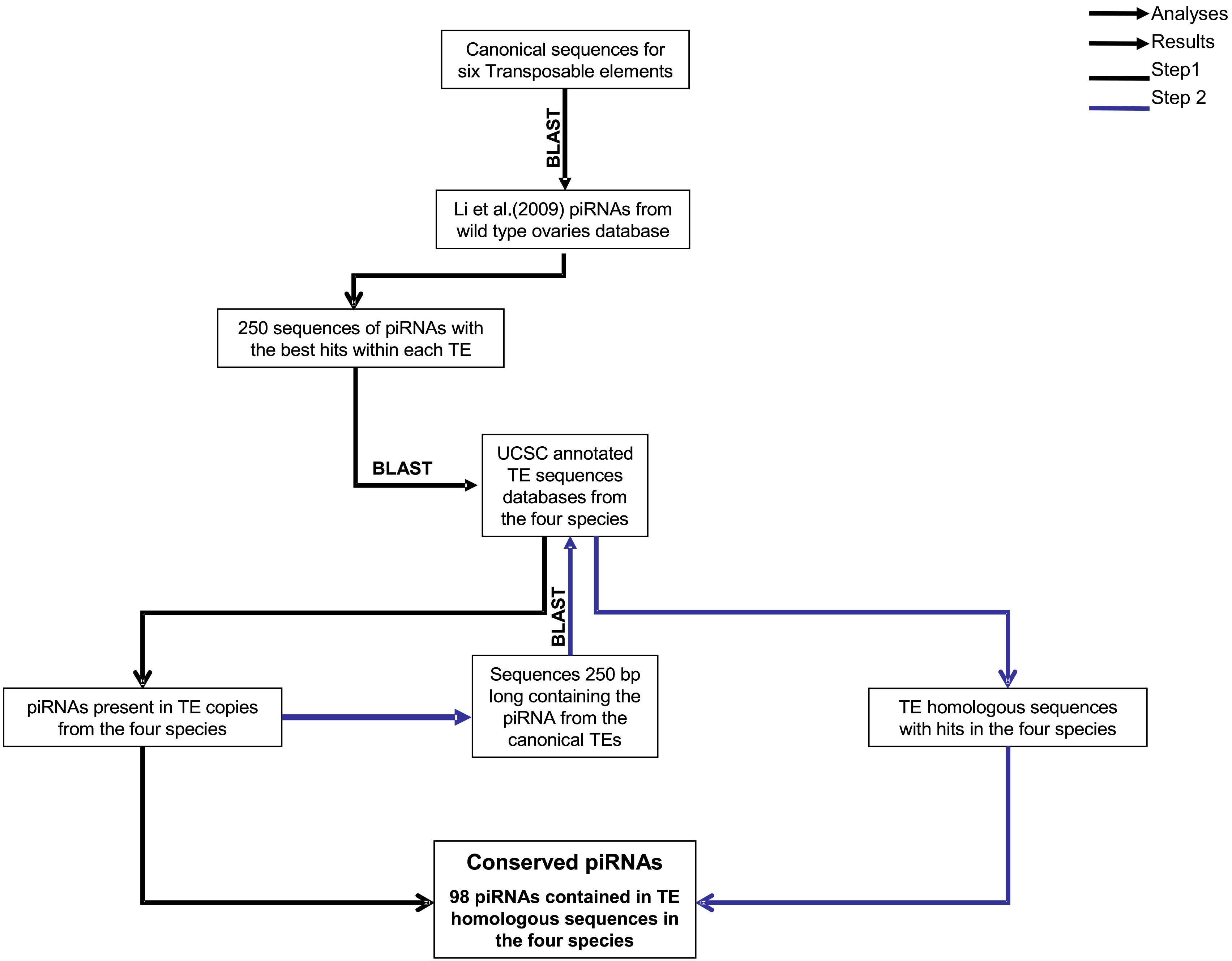

Figure S10. Diagram of the approach to find conserved piRNA targets in TEs among copies and species. Different color arrows indicate two different and alternative steps. Step 1 (black arrows) piRNA target sequences from the database of each annotated TE in all four species were detected. Step 2 (blue arrows) homologous TE sequences containing the piRNA target sequence were obtained. Conserved piRNAs are that present in all four species and within a homologous canonical TE sequence.
