# Supplementary material for: HeT-A_pi1, a piRNA Target Sequence in the Drosophila Telomeric Retrotransposon HeT-A, Is Extremely Conserved across Copies and Species
Source: PLoS One. 2012 May 21;7(5):e37405. doi: 10.1371/journal.pone.0037405 (PMC3357415; doi:10.1371/journal.pone.0037405)
Supplement: Figure S11 — Conservation index of the fifteen highly conserved piRNA target sequences in the different Drosophila species. (PDF) [file pone.0037405.s011.pdf]

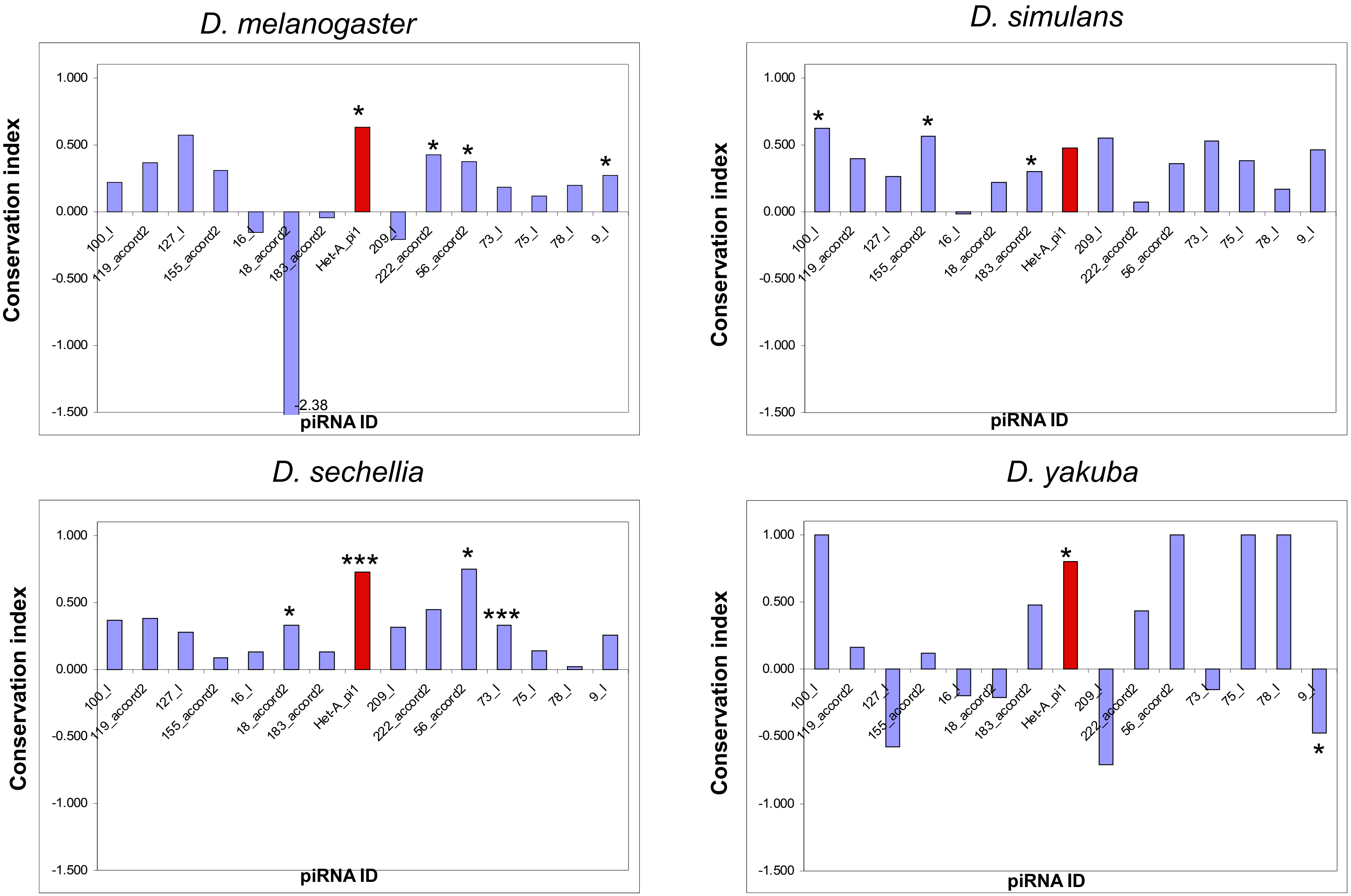

Figure S11. Conservation index of the fifteen highly conserved piRNA target sequences in the indicated species. The HeT-A_pi1 target labeled in red. Positives and negative bars indicate higher or lesser conservation of the piRNA target sequence in respect to the TE sequence where the target is embedded. Stars indicate cases where the number of nucleotide changes of the piRNA target sequence is significantly different than the TE sequence where the target is embedded (*=p<0.05; ***=p<0.005)
