# Supplementary material for: HeT-A_pi1, a piRNA Target Sequence in the Drosophila Telomeric Retrotransposon HeT-A, Is Extremely Conserved across Copies and Species
Source: PLoS One. 2012 May 21;7(5):e37405. doi: 10.1371/journal.pone.0037405 (PMC3357415; doi:10.1371/journal.pone.0037405)
Supplement: Figure S12 — Mean values of conservation index (red) and constraints (blue) of the fifteen highly conserved piRNAs target sequences among D. melanogaster, D. simulans, D. sechellia and D. yakuba species. (PDF) [file pone.0037405.s012.pdf]

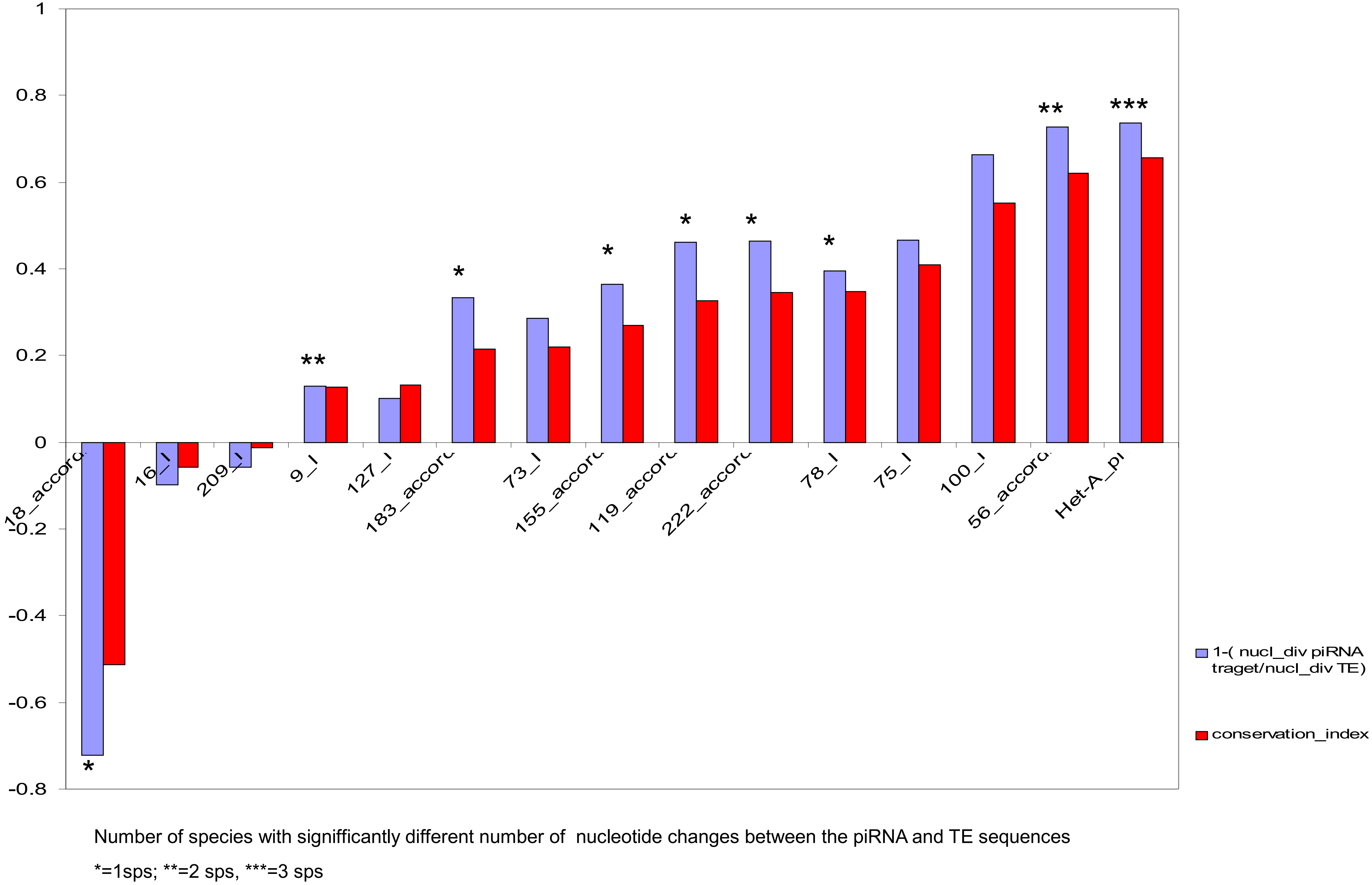

Figure S12. Mean values of conservation index (red) and constraints (blue) of the fifteen highly conserved piRNAs target sequences among *D. melanogaster, D. simulans, D. sechellia* and *D. yakuba* species. Asterisks label those cases (species) where the piRNA target sequence has significantly different number of nucleotide changes than the flanking sequence:
