# Supplementary material for: HeT-A_pi1, a piRNA Target Sequence in the Drosophila Telomeric Retrotransposon HeT-A, Is Extremely Conserved across Copies and Species
Source: PLoS One. 2012 May 21;7(5):e37405. doi: 10.1371/journal.pone.0037405 (PMC3357415; doi:10.1371/journal.pone.0037405)
Supplement: Methods and Results S1 — (DOC) [file pone.0037405.s020.doc]

**Petit et al. “**HeT-A_pi1, a piRNA target sequence in the Drosophila telomeric retrotransposon *HeT-A*, is extremely conserved across copies and species**”**

**Supplementary Methods**

***Sequence alignments and analyses of conservation and diversity*.** All alignments were obtained using Muscle3.6 software [1]. Estimates of nucleotide diversity among elements and species were obtained and tests of conservation of fragments of the element were performed using DNAspv5 [2].

Nucleotide diversity was measured as the average number of pairwise nucleotide differences among all the sequences in a given analysis, which in some cases includes sequences coming from different copies, different species or both. Conservation estimates were obtained as C = 1 – P, where P is the proportion of nucleotide changes. Conservation testing was performed by means of the test implemented in DNAspv5, which computes the probability of the observed or fewer changes within any given region using an hypergeometric distribution and the conservation level, C, of the whole fragment under analysis [2].

To find conserved regions along the *HeT-A* element we defined a conservation threshold (CT) and a minimum window length (MWL) based on the number of observed nucleotide changes in the sequences as MWL=79 and CT=0.76, using the option “Dynamic defined parameters given the observed S“ in DNAspv5 [2]. Additionally and for completeness, we investigated sequence conservation with three different MWLs and CTs: (1) MWL=75 and CT=0.8; (2) MWL=50 and CT=0.9 and (3) MWL=25 and CT=1.

***Correlation analysis between number of piRNAs and nucleotide diversity.*** Correlation analyses between the number of piRNAs matching the sequences of the *HeT-A* copies and their nucleotide diversity levels (measured as the average number of pairwise differences among *HeT-A* copies) were performed in two sets of non-overlapping windows of 100 and 250 nucleotides. The number of piRNAs targets within each window was estimated as the count of *HeT-A* 30 ntds overlapping fragments matching different RNA reads from Li et al [3] database. Nucleotide diversity was estimated for each window using DNAspv5, and windows for which this estimate could not be obtained (*i.e.* windows with large indels) were excluded. For other elements, the number of piRNAs targets was obtained in the same way than for *Het-A*, using the canonical sequence of each element. Nucleotide diversity was estimated with alignments with homologous long sequences from databases of each element. In all cases nucleotide diversity was calculated based on 5 random sequences

***Distribution of piRNAs along the HeT-A sequence.*** The unequal distribution of the number of piRNAs matching the different functional parts of the element (the *gag* coding region and the 3’ and 5’ UTRs) was tested by means of a chi square test in which the expected numbers of piRNAs was proportional to the length of the functional parts in each *HeT-A* copy.

**Supplementary Results**

**Sequence conservation in the 3’UTR of *HeT-A***

Our first approach to investigate the nucleotide conservation of the *HeT-A* sequence consisted in searching for regions with high nucleotide identity among the six *D. melanogaster* complete *HeT-A* elements that have been described [4]. Nucleotide diversity along the element is showed in Figure 1A (Main Text). The result of the analysis of sequence conservation in 25 ntds windows showed only three small (no longer than 100 ntds) fully conserved regions (Figure 1A and supplementary Table S1). These three regions (labeled R1-R3) are within the 3’UTR of the elements (Supplementary Table S1). To find sequence conservation among elements from different *Drosophila* species we performed blast analyses of the *HeT-A* copy (*HeT-A*{}6268) against the *HeT-A* databases for eleven *Drosophila* species with sequenced genomes (obtained from [UCSC Genome Bioinformatics](http://genome.ucsc.edu/) website). Supplementary Table S2 shows the number of significant hits obtained for each functional part of the element in each *Drosophila* species. The 5’ UTR is the least represented region, with significant hits obtained only in *D. melanogaster*. The nucleotide sequence of the *gag* coding region was only found among closest species (*i.e.* the melanogaster subgroup). The 3’UTR is the most abundant sequence with significant hits in *D. melanogaster*. Interestingly, this sequence seems to be abundant also in the other species including *D. yakuba* where no other homology was found (Supplementary Table S2).

More than 50% of the 3’UTR hits are within the last 500 ntds of the element, with the two significantly conserved regions, R2 and R3 (Figure 1A). Sequence conservation among *HeT-A* orthologue copies was estimated for the last 500 ntds of the 3’UTR performing an alignment with sequences longer than 350 ntds obtained from the previous blast analysis (Supplementary Table S2). Figure 1B corresponds to a sliding window analysis of nucleotide conservation/diversity among homologous *HeT-A* sequences from the 3’UTR of different species of the *melanogaster* group. Conservation analysis of this alignment shows that regions R2 and R3 are also significantly conserved among species (R2: conservation=0.526, p-value = 0.0103, length = 39 bp; R3: conservation=0.597, p-value = 0.000, length = 75bp; Figure 1B, and supp. Figure S1).

**Function of the *HeT-A* 3’UTR conserved regions (R2 and R3)**

To look for putative functions of the detected conserved regions R2 and R3 we performed a blast analysis of the 3’UTR end sequence from the *HeT-A*{}6268 copy (500 ntds), which contains the conserved regions, against the nucleotide database at NCBI. This analysis revealed homology with two mapped *D. melanogaster* cDNAs (BT015972 and BT030306) and one antisense piRNA from *D. melanogaster* testes (AB297338.1, see [5]. The annotated transcripts of both cDNAs are antisense to *HeT-A*. The cDNA BT030306 maps to a complete copy of the unassembled fraction of the *D. melanogaster* genome. The cDNA BT015972 has three complete copies that map in the genome (two at the left telomere of chromosome X: 1800..3991; 16447..18642 and one at the right telomere of chromosome 4: 1300963..1303158). The high identity (27/28) of the R2 conserved region with the antisense piRNA sequence, AB297338.1 (Figure 1C) suggests that the R2 conserved region corresponds to the target sequence for this piRNA. The search of the sequence of piRNA AB297338.1 in the database of piRNAs from *D. melanogaster* wild-type ovaries obtained by Li et al. [3] discovered 3 reads containing an identical 28 ntds long sequence. Seven reads of the same sequence were also found in the database of piRNAs bound to the Piwi protein [3].

**Distribution of piRNA target sequences along the *HeT-A* element**

The 7,048 different putative piRNA sequences matching different *HeT-A* copies in *D. melanogaster* have an unequal distribution along the different functional parts of the element. The average number of piRNA targets is significantly higher than expected from sequence length inside the *gag* coding region (~2800bp, N expected ~557, observed ~770) and lower in the 5’ (~800bp, N expected ~159, observed ~101) and 3’ UTRs regions (~2300 bp, N expected ~429, observed ~303) (Chi-Square test, all p-values<10^-6^), indicating that the sequence of the *gag* coding region is the main piRNA target in the different *HeT-A* copies (Supplementary Figure S3A and Table 1). Since the *gag* coding region is quite conserved among copies of the element (TableS1 and Figure 1A) we found that nucleotide diversity estimates among the *HeT-A* sequences and the number of their corresponding piRNAs are negatively correlated (Table S3), as shown in Figures S2A and B (see Supplementary Methods). Also, the correlation was significant for the case in which the search of piRNAs was relaxed to an exact match of 15 nucleotides (Table S3), indicating that the correlation is not caused by the strategy we used to look for piRNA target sequences. In contrast, the correlation vanishes when it is estimated without the sequence windows containing the gag coding region.(Figures S3 A and B). From the point of view of the cell, in its arms race with the retroelements, it would make sense to locate piRNA targets in conserved regions of the TEs, since that would ease their control. However, a correlation study with elements *I*, *copia*, *gypsy* and *accord2* show there is no overall relationship between the conservation of a site and the amount of piRNAs that are detected there (Figures S4-S7). Thus, the significant correlation found in *HeT-A* is driven by the piRNAs targeting gag coding regions, that is the *gag* coding region is targeted by more piRNAs that other, less conserved, parts of the element. This excess of the piRNAs targeting the gag coding region sequence could be produced just because active elements that are retrotransposed to the end of the chromosome have complete gag coding regions and possibly are the source of the primary transcripts that will produce piRNAs sequences.

The frequency distribution of the piRNA target sequences among the six copies of the *HeT-A* element is presented in Figure S9, and it shows that is expected under a arms-race scenario. That is, most of piRNAs target a single copy of *HeT-A* , while a very few of them target all *HeT-A* copies.

**Transcript analyses**

The 3’UTR transcripts were grouped by homology (number of pairwise nucleotide differences < 3) and mapped by Blast onto the *D. melanogaster* reference genome. Ten different groups of transcripts were defined (marked with different colors in Supplementary Table S5). Seven groups map with more than 97% of identity within the arrays of telomeric elements in the two completely assembled telomeres of *D. melanogaster* (4R and XL). We also estimated the average number of pairwise differences between each transcript and the six complete copies of *HeT-A* in *D. melanogaster* finding that one of the cloned transcripts from ovaries (ov11) shows 100% identity with the *HeT-A*{}6274 *HeT-A* copy. Copies 4R6274 and 23Znk are the two complete *HeT-A* elements with a higher number of small RNA reads (Table 1, Main Text), suggesting that these two master copies are maybe among the most active in *D. melanogaster.* In fact, experimental evidence (data not shown) shows that the *HeT-A* element known as *HeT-A*23Znk [6] is one of the most actively transcribed *HeT-A* copies.

**Conserved piRNAs target sequences in other transposable elements**

The conservation of piRNA target sequences was analyzed for six other transposable elements with evidence of control by piRNAs: *gypsy1*, *accord*-2, *jockey*, *TART-B1*, *I* and *copia* [3, 7]. The *D. melanogaster* canonical sequence of each one of these six transposable elements was obtained from *Flybase*. For each element and species (*D. melanogaster*, *D. simulans*, *D. sechellia* and *D. yakuba*) a blast-database (TE Database) was constructed with the set of annotated TE sequences obtained from the UCSC Genome Bioinformatics site, in the same way as for *HeT-A* (see main text).

In the first step of the analysis the *D. melanogaster* canonical sequence of each one of the six transposable elements was blasted against the small-RNA reads database from Li et al. [3];(see Methods). The sequences of the 250 better hits for each element were blasted against the corresponding Database of TEs for each species, with the algorithm modified for small sequences (Supplementary Figure S10, black lines). This step of the analysis discovered 219 small RNA reads with significant hits in all four species (*gypsy1*=90, *I*=39, *copia*=12, *jockey*=2, *TART-B1*=29). The redundant piRNAs were eliminated obtaining a list of 117 putative piRNA target sequences present in all four species TE dbs.

The second step of the analysis consisted in extracting a 250 ntds long sequence from the canonical sequence of each element, starting 100 ntds before the beginning of each piRNA with significant blast hit in the four species TE Databases. These 250 ntds long sequences were blasted against the corresponding TE Database, with the default algorithm of Blast (Supplementary Figure S10, blue lines). This step of the analysis allowed us to obtain TE sequences with homology to a 250 bp long sequence of the canonical element that does not necessarily contain the piRNA sequence. After crossing this list with the one obtained in the first step, we end-up with a total of 98 putative piRNAs targets whose sequences and surrounding region are conserved in the four species (*accord2*=32, *copia*=6, *gypsy1*=30, I=22, *TART-B1*=7, *jockey*=0. The proportion of the homologous TE sequence (hits of the 250 ntds sequence) containing the piRNA target sequence in each species is presented in Supplementary Table S6. Only 15 of these piRNA target sequences are highly conserved in all four species, meaning that more than 50% of TE homologous sequences contain the piRNA target sequence (Table S7, Figure S10).

The elements with higher number of conserved piRNA target sequences among species belong to the Gypsy family (*gypsy1*: 12% and *accord2*: 13%). For *gypsy1* none of the conserved piRNA targets was present in more than the 50% of the TE homologous sequences in all species; while for *accord2* nearly 3% of the piRNA target sequences are highly conserved, probably reflecting differences in the dynamics of piRNAs controlling these retrotransposons [3].

Other elements present fewer conserved piRNA targets. For the elements related to *HeT-A* (LINE type elements: *TART-B1* and *jockey*) only 3% of the piRNA target sequences seem to be moderately conserved in the four species. *TART-B1* targets are never present in more than 50% of TE sequences in all species. There are no conserved piRNA targets for *jockey*. Finally, the two Group I TEs unrelated to *HeT-A* [3] *copia* (LTR retrotransposon) and *I* (LINE element) presented different features. *Copia* contains 2,4% of moderately conserved piRNA target sequences; while *I* harbors a total of 9% of conserved piRNA target, out of which 3% are highly conserved.

**References**

1. Edgar RC: **MUSCLE: multiple sequence alignment with high accuracy and high throughput**. *Nucleic acids research* 2004, **32**(5):1792-1797.

2. Librado P, Rozas J: **DnaSP v5: a software for comprehensive analysis of DNA polymorphism data**. *Bioinformatics (Oxford, England)* 2009, **25**(11):1451-1452.

3. Li C, Vagin VV, Lee S, Xu J, Ma S, Xi H, Seitz H, Horwich MD, Syrzycka M, Honda BM *et al*: **Collapse of germline piRNAs in the absence of Argonaute3 reveals somatic piRNAs in flies**. *Cell* 2009, **137**(3):509-521.

4. George JA, DeBaryshe PG, Traverse KL, Celniker SE, Pardue ML: **Genomic organization of the Drosophila telomere retrotransposable elements**. *Genome research* 2006, **16**(10):1231-1240.

5. Nishida KM, Saito K, Mori T, Kawamura Y, Nagami-Okada T, Inagaki S, Siomi H, Siomi MC: **Gene silencing mechanisms mediated by Aubergine piRNA complexes in Drosophila male gonad**. *RNA (New York, NY* 2007, **13**(11):1911-1922.

6. Danilevskaya ON, Arkhipova IR, Traverse KL, Pardue ML: **Promoting in tandem: the promoter for telomere transposon HeT-A and implications for the evolution of retroviral LTRs**. *Cell* 1997, **88**(5):647-655.

7. Lu J, Clark AG: **Population dynamics of PIWI-interacting RNAs (piRNAs) and their targets in Drosophila**. *Genome research* 2010, **20**(2):212-227.
